# Genetic Mapping Identifies Novel Highly Protective Antigens for an Apicomplexan Parasite

**DOI:** 10.1371/journal.ppat.1001279

**Published:** 2011-02-10

**Authors:** Damer P. Blake, Karen J. Billington, Susan L. Copestake, Richard D. Oakes, Michael A. Quail, Kiew-Lian Wan, Martin W. Shirley, Adrian L. Smith

**Affiliations:** 1 Institute for Animal Health, Compton, Berkshire, United Kingdom; 2 Pathology and Infectious Diseases, Royal Veterinary College, University of London, North Mymms, United Kingdom; 3 Wellcome Trust Sanger Institute, Wellcome Trust Genome Campus, Hinxton, United Kingdom; 4 Malaysia Genome Institute, UKM-MTDC Technology Centre, Selangor, Malaysia; 5 School of Biosciences and Biotechnology, Faculty of Science and Technology, Universiti Kebangsaan Malaysia, Selangor, Malaysia; 6 Department of Zoology, University of Oxford, Oxford, United Kingdom; Washington University School of Medicine, United States of America

## Abstract

Apicomplexan parasites are responsible for a myriad of diseases in humans and livestock; yet despite intensive effort, development of effective sub-unit vaccines remains a long-term goal. Antigenic complexity and our inability to identify protective antigens from the pool that induce response are serious challenges in the development of new vaccines. Using a combination of parasite genetics and selective barriers with population-based genetic fingerprinting, we have identified that immunity against the most important apicomplexan parasite of livestock (*Eimeria* spp.) was targeted against a few discrete regions of the genome. Herein we report the identification of six genomic regions and, within two of those loci, the identification of true protective antigens that confer immunity as sub-unit vaccines. The first of these is an *Eimeria maxima* homologue of apical membrane antigen-1 (AMA-1) and the second is a previously uncharacterised gene that we have termed ‘immune mapped protein-1’ (IMP-1). Significantly, homologues of the AMA-1 antigen are protective with a range of apicomplexan parasites including *Plasmodium* spp., which suggest that there may be some characteristic(s) of protective antigens shared across this diverse group of parasites. Interestingly, homologues of the IMP-1 antigen, which is protective against *E. maxima* infection, can be identified in *Toxoplasma gondii* and *Neospora caninum*. Overall, this study documents the discovery of novel protective antigens using a population-based genetic mapping approach allied with a protection-based screen of candidate genes. The identification of AMA-1 and IMP-1 represents a substantial step towards development of an effective anti-eimerian sub-unit vaccine and raises the possibility of identification of novel antigens for other apicomplexan parasites. Moreover, validation of the parasite genetics approach to identify effective antigens supports its adoption in other parasite systems where legitimate protective antigen identification is difficult.

## Author Summary

Protozoan parasites are responsible for serious diseases in humans and livestock species. Vaccination is a declared intervention of choice with these infections, but even after many years of effort few effective vaccines are available. Identification of the right antigens for inclusion in sub-unit vaccines is a particular problem with complex pathogens. Moreover, the host response does not discriminate between protective and non-protective antigens, confounding development of effective screening systems. This study represents the culmination of work using parasite genetics and immunity as a selective barrier to find parts of the parasite genome targeted by immunity. The pathogen used in these studies (*Eimeria maxima*) is very important in livestock and related to a number of human pathogens including those responsible for malaria. Our studies indicate that just six regions in the genome were targeted by immunity and two of these have now been interrogated to determine the protective antigen encoding gene. Interestingly, one of these (called AMA-1) has homologues known to be protective with other apicomplexan parasites. This raises the intriguing possibility that a set of homologous antigens may be protective across the apicomplexan parasites and that protective antigen discovery in one parasite may generate new leads in other vaccine programmes.

## Introduction

The protozoan phylum Apicomplexa contains pathogens of substantial medical and veterinary importance including *Plasmodium*, *Toxoplasma*, *Cryptosporidium*, *Eimeria*, *Neospora* and *Theileria* species. Despite several decades of effort vaccines protective against these and other related parasites are scarce. Empiric approaches to identify genuinely immunoprotective antigens as vaccine candidates have achieved mixed results (e.g. [1], [2]) and problems differentiating immunogenicity from real immune protection persist. Here we report the culmination of our efforts to develop a new approach to candidate antigen identification, one based upon using immunity as a selective barrier allied with pathogen genetics and mapping to identify true immune-targeted loci. Having identified candidate genomic regions we used a variety of strategies to locate the antigen responsible for protection. This approach has yielded new protective antigens for *Eimeria maxima*, one of the most important apicomplexan parasites to afflict livestock and provides insight into the nature of protective antigens in a broader context.

As sustainable food security gains in importance pathogens which impact upon poultry production are re-emerging as serious threats to global food supply and human poverty [3], [4]. *Eimeria* species parasites have a globally enzootic distribution and can cause severe enteric disease in all livestock, most notably poultry, where the annual cost is estimated to exceed £2 billion worldwide [5]. Current control is dominated by prophylactic application of anticoccidial drugs but drug resistance, political/consumer concerns over residues and the lack of new pipeline products renders this an unsustainable approach. Alternatives are limited by cost and/or efficacy and new solutions are urgently required.

*Eimeria* are highly immunogenic parasites. Infection with as few as five *E. maxima* oocysts can induce complete protective immunity against subsequent homologous challenge [5]. Conversely, different strains of *E. maxima* can be antigenically diverse such that infection by one strain can induce little or no protection against challenge by a different strain [6]. Through the combination of *in vivo* selection imposed by strain-specific immunity and/or anti-parasitic medication with a population-based mapping strategy developed for use with apicomplexan parasites, we and others have shown that loci affecting strain-specific immunity can be mapped genetically [6], [7], [8]. We previously identified a panel of genetic markers within the *E. maxima* Weybridge (W) genome whose inheritance correlated absolutely with susceptibility to strain-specific immune killing [6]. Here we identify that the genetic markers associated with immunity map to just six regions representing less than 0.8% of the genome. Using a combination of sequencing, fine mapping and vaccination screens we have identified antigens responsible for protection in two of these loci. Homologues of both can be found in multiple apicomplexan parasites and one is known to be protective in diverse parasites of this phylum including *Plasmodium* spp. [1]. Hence, the novel antigens and remaining immune-mapped loci found within this study will have direct impact on eimerian vaccine development and have potential to impact on development of new vaccines with other apicomplexan parasites.

## Results/Discussion

**Figure 1:**
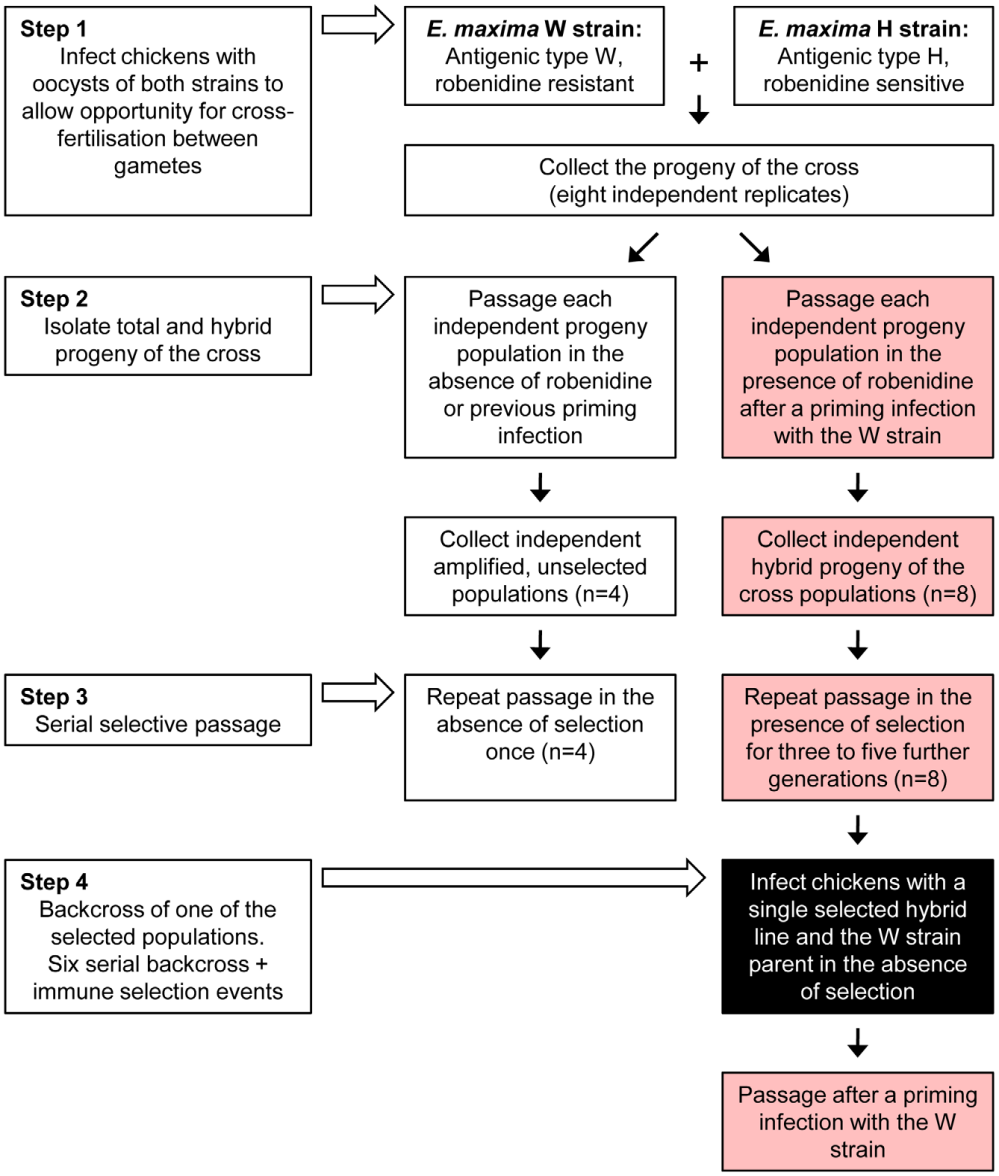
Production and selection of the parasite mapping panel. All parasite populations shown here were included in the DNA mapping panel with the exception of those denoted in the black-filled box. All populations shown in the red filled boxes were subjected to immune selection (± dietary robenidine selection). The number of replicates per population is indicated in each box.

**Figure 2:**
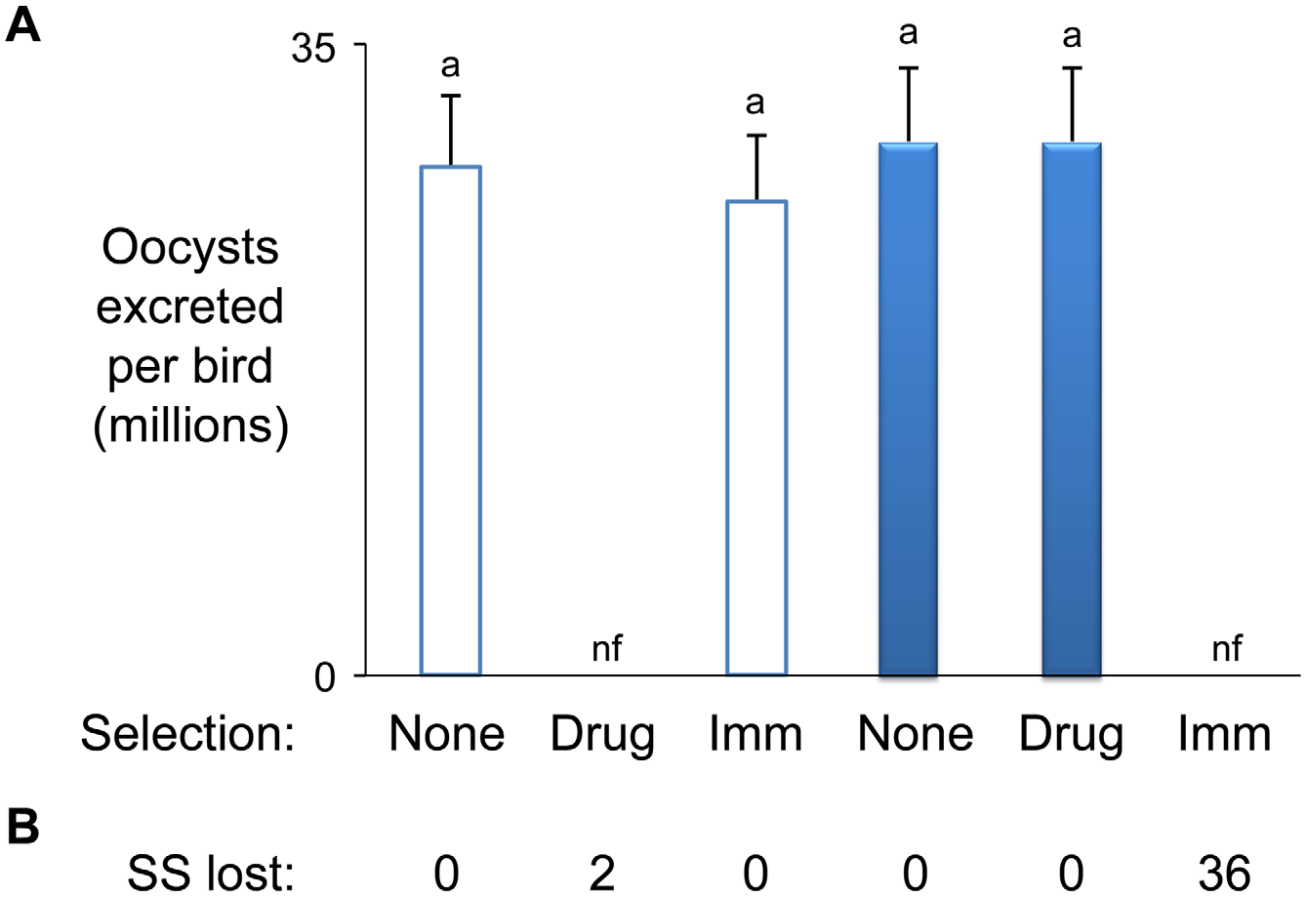
*Eimeria maxima* strain-specific traits. (**A**) Phenotypes. The influence of drug (robenidine) and W strain-specific immunity on H and W strain parasite replication reveals polymorphic strain-specific selectable traits (H = hollow bars, W = solid bars). (**B**) Genotypes. The H and W strains were found to be defined by 588 and 534 strain-specific (SS) AFLP markers respectively. Comparison of AFLP marker profiles amplified from both parents and the progeny of each independent cross between the H and W strains before and after passage under combined drug and W-specific immune selection revealed the reproducible loss of between 0 and 36 strain-specific markers. nf = no oocysts found.

The *E. maxima* Houghton (H) and W strains are genetically and phenotypically distinct [6]. The H strain is characterised by sensitivity to dietary robenidine at 66 ppm and complete escape from W strain-specific immune killing during passage in inbred Line C White Leghorn chickens (induced by previous host exposure to the W strain). In contrast, the W strain is resistant to 66 ppm robenidine but completely susceptible to W strain-specific immune killing. Genetic characterisation of the *E. maxima* H and W strains using amplified fragment length polymorphism (AFLP) with five different enzyme combinations to minimise restriction-associated bias generated a total of 3,230 genetic markers (**Table S1**). Comparison between the two strains revealed 1,122 markers to be polymorphic (34.7%; considerably higher than described previously for two *Eimeria tenella* strains [9]). Inheritance patterns of these strain-specific (SS) markers by a parasite mapping panel (**Figure 1**; **Table S2**), which included the uncloned progeny of eight independent H/W strain crosses before and after concurrent immune/robenidine selection, revealed the absolute correlation of 36 and 2 markers with the immune and drug barriers respectively (**Figure 2**). Importantly, all of the 36 immune correlated markers were subject to strong negative selection and were completely lost in all independent parasite lineages. Some other AFLP fragments were found to be less intensely amplified from immune selected parasites, suggesting more distant linkage to a mapped locus or linkage to loci containing genes contributing subtly to the complex biology of protection, either as modifiers, regulators, or minor antigens. Our analysis has focussed on the regions marked by AFLP fragments under the strongest selection by immunity. Serial *in vivo* passage of four hybrid populations under double barrier selection for up to five generations did not change the marker inheritance profile (as [6]).

Sequencing the 36 SS markers whose inheritance correlated with susceptibility to strain-specific immune selection identified 32 suitable for use as hybridisation probes (EMBL FN813211-8 and unpublished). When radiolabeled with ^32^P and used to probe an *E. maxima* W strain BAC library representing ∼7.5-fold genome coverage a total of nine BACs were highlighted (**Table 1**). Each BAC was identified by at least two independent markers. BAC end sequencing and subsequent sequence-specific PCR facilitated the assembly of these nine BACs into six clusters, representing six distinct loci (**Table 1**). BAC insert release by *Not* I digestion and resolution by pulsed field gel electrophoresis (PFGE) provided approximate cluster sizes which were later refined following BAC insert sequencing and assembly (**Table 1**; FN813242–4 and unpublished). Marker hybridisation to Southern blotted full PFGE-resolved karyotypes identified the chromosomal location of five of the six clusters (**Table 1**).

**Table 1:** Summary of loci mapped within the *Eimeria maxima* W strain genome associated with susceptibility to strain-specific immune killing.

Locus	No. of	No. of	∼Mapped BAC size (Kb) (% genome*)		Chromosome	% protection conferred**		
	Markers	BACs	Before backcross	After backcross		BAC-1	BAC-2	BAC-3
1	3	1	127 (0.21)	45 (0.08)	nd	**16.0±3.1** a		
2	13	2	268 (0.45)	78 (0.13)	13–14	1.3±1.4	**43.6±5.3** b	
3	7	3	350 (0.58)	90 (0.15)	3–4	−2.5±1.9	**17.1±2.2** a	2.7±1.7
4	4	1	114 (0.19)	85 (0.14)	5	**37.3±2.3** a		
5	2	1	90 (0.15)	84 (0.14)	12–14	**59.7±6.8** b		
6	3	1	145 (0.24)	80 (0.13)	12–14	1.9±1.1		
Total	32	9	1094 (1.84)	462 (0.77)	3–6			
Average	5	2	182 (0.30)	77 (0.13)	-			

*Based upon a predicted 60 Mb genome.

**Immune protection associated with each mapped BAC was measured as the oocyst output resulting from W strain challenge following immunisation by infection with the heterologous H strain transiently transfected with one candidate BAC compared to transfection with a randomly selected control BAC. Comparison between the latter and unimmunised birds was not significantly different (5.4±1.6). The average of three replicate transfections performed on separate occasions is shown. nd = not done.

a Significantly different (p<0.01),

b significantly different (p<0.005), using ANOVA+Tukey's post-hoc.

Confirmation of the potent immunogenicity of the early stages of the *E. maxima* lifecycle supported the development of an *in vivo* BAC-screening system to begin fine mapping for each locus (**Table S3**, trial 1). Utilising recent advances in transfection protocols for *Eimeria* [10] purified *E. maxima* H strain sporozoites were transiently transfected with each individual W strain BAC identified by hybridisation to an immune-correlated SS marker or a randomly selected control BAC (**Table S3**, trial 2). When Line C chickens were immunised by infection with 1×10^6^ test-, control- or non-BAC transiently transfected H strain sporozoites, drug-cleared by exposure to 66 ppm dietary robenidine to avoid clinical coccidiosis and challenged three weeks later with the W strain the capacity to induce cross-protective immunity was conferred by BACs derived from five of the six mapped loci (**Table 1**). In three independent experiments BACs representing loci 2 and 5 consistently induced the highest levels of immune protection, followed by BACs from loci 1, 3 and 4 (**Table 1**). No cross-protective phenotype was observed using the sixth locus, suggesting either a mapping error, a requirement for a strain-specific partner molecule (encoded elsewhere in the genome) or stage-specific antigen expression towards the latter stages of the *E. maxima* lifecycle. The transient nature of our transfection approach is such that that if a protective antigen or other protection-affecting element was not expressed before loss of the host BAC then protection would not be detected. The six loci under intense immune selection (i.e. essential loci) are dispersed across the *E. maxima* genome (**Table 1**). Importantly, BAC-transfection based vaccination with five of the loci induced strain-specific immunity, indicating that each can act independently. Having identified relevant polymorphisms we can use this information to perform much wider studies on the genetic basis of protection including the population dynamics of the protective-antigen encoding genomic regions in laboratory and field populations. Preliminary co-transfection trials using two BAC clones representing loci 2 and 3 induced a higher level of immune protection (83.2±7.7% referenced to control BAC transfected parasites) than either individual BAC (43.1% and 17.1% protection respectively). Indeed, locus 2/3 co-transfection immunisation gave greater protection than the additive effects of the individual loci; future studies will explore the combinatorial nature of protection in more detail.

**Figure 3:**
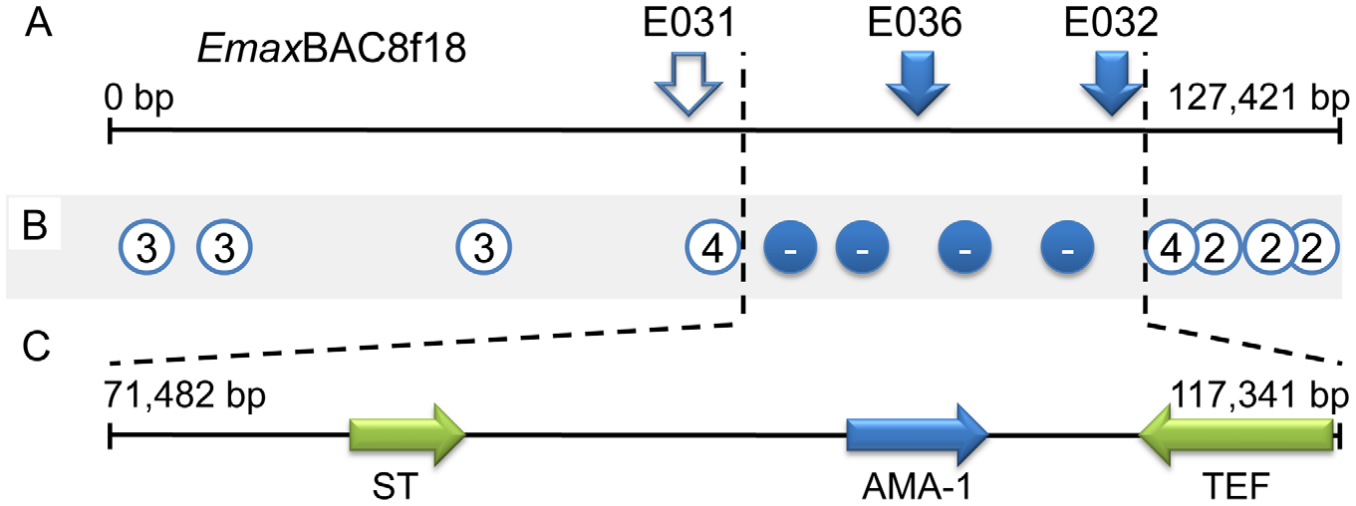
Fine mapping locus 1 correlated with susceptibility to strain-specific immune killing. (**A**) The distribution of strain-specific AFLP markers lost under immune selection across BAC *Emax*BAC8f18. Solid markers remained absent after six generations of backcrossing under selection, hollow marker E031 reappeared in backcrossed generation four. (**B**) Recovery of strain-specific markers by immune selected backcross generations 1–6, the number represents the generation at which the marker first reappeared, − = did not reappear. (**C**) Transcribed sequences identified within locus 1 (ST = putative sulphate transporter, AMA-1 = apical membrane antigen 1 homologue, TEF = putative transcription elongation factor).

**Figure 4:**
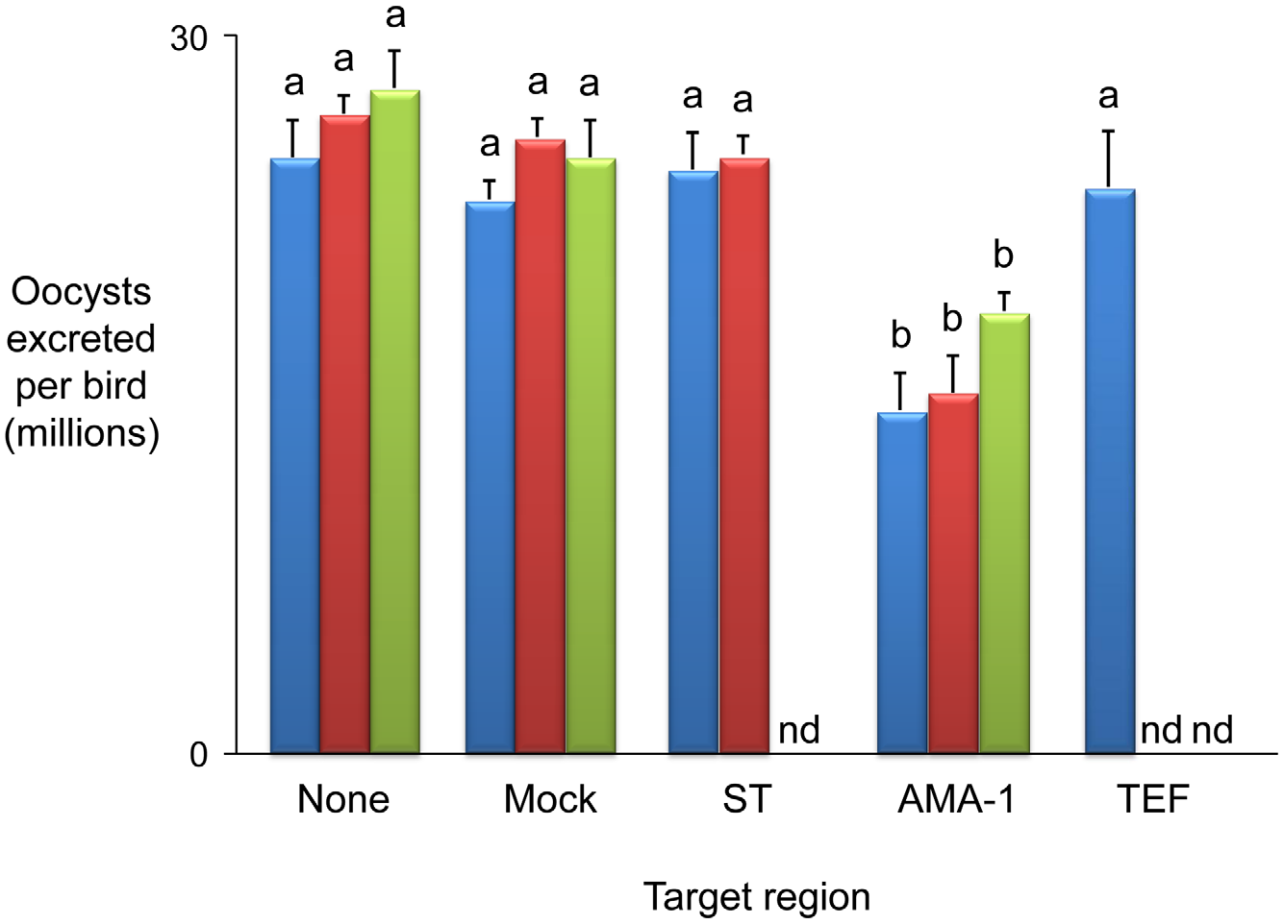
Immunising ability of individual genes identified within immune mapped locus 1. Average oocyst output per bird following *Eimeria maxima* W strain challenge of birds not previously immunised (none), mock immunised (mock) or immunised using the putative sulphate transporter (ST), apical membrane antigen-1 (AMA-1) or transcription elongation factor (TEF) mapped antigens. Blue bars indicate candidates presented as *E. maxima* H strain vectored transgenes (mock = *E. maxima* H strain transfected in the absence of transgene DNA). Dark red bars indicate candidates presented as DNA vaccines (mock = empty vaccine vector). Green bars indicate candidates delivered as purified, *E. coli*-expressed, thioredoxin-tagged recombinant proteins (mock = thioredoxin only). nd = not done. Bars marked with different letters were significantly different within each immunisation strategy (p<0.05).

The parasite mapping panel was supplemented by backcrossing and re-selecting one double barrier-selected population to the immune-targeted parental W strain on six successive occasions. Genotyping each selected backcross generation maintained linkage with 11/32 SS markers, covering all six loci (e.g. **Figure 3A**). Backcross genotyping fine-mapped locus 1 to ∼64 Kb of genomic sequence contained within BAC *Emax*BAC8f18. Typing additional SS PCR markers produced by targeted sequencing from the H strain across locus 1 provided further focus, mapping the region of interest to ∼50 Kb (**Figure 3B**, **Tables S4** and **S5**). Bioinformatic examination of fine-mapped locus 1 identified three predicted coding regions, annotated as encoding (i) a sulphate transporter, (ii) an apical membrane antigen-1 (AMA-1) homologue and (iii) a transcription elongation factor (**Figure 3C**). Sequencing from the H strain identified amino acid polymorphism in the first two candidates but not the third (FN813219–24). *In vivo* infection by H strain sporozoites transiently transfected with purified genomic Long Distance (LD) PCR amplicons covering each predicted coding sequence and flanking regions induced a cross-protective immune phenotype only when using *EmAMA-1* (**Figure 4**). AMA-1 has been widely proposed as an anti-apicomplexan vaccine candidate [11], [12] and the *E. tenella* homologue has recently been found to be similarly protective (Tomley, Billington *et al*, manuscript in preparation). Immunisation using *EmAMA-1* as a DNA vaccine in the eukaryotic expression vector pcDNA3.1(+) (Invitrogen) or as a bacterially-expressed recombinant protein induced significant immune protection against subsequent challenge by the W strain (**Figure 4**). The level of immune protection observed following immunisation using *EmAMA-1* as a DNA vaccine was similar when challenged with 250 or 2,000 sporulated oocysts (data not shown). Comparison between the H and W *EmAMA-1* coding sequences revealed four nucleotide polymorphisms, two of which yield non-synonymous changes, one in the likely pro-domain and one in domain 1 [13]. Interestingly, AMA-1 domain 1 is also polymorphic among *Plasmodium falciparum* strains and polymorphism in this region confers the greatest level of escape from inhibitory antibodies [14]. Immunity induced by exposure to *Eimeria* spp. is highly species specific and although the *E. maxima* and *E. tenella* AMA-1 molecules share many structural characteristics the primary amino acid sequence is considerably different (57% aa identity). This level of divergence between eimerian AMA-1 is similar to that observed with *Plasmodium* AMA-1 from different species (e.g. *P. falciparum* compared with *Plasmodium vivax*, 60% aa identity).

**Figure 5:**
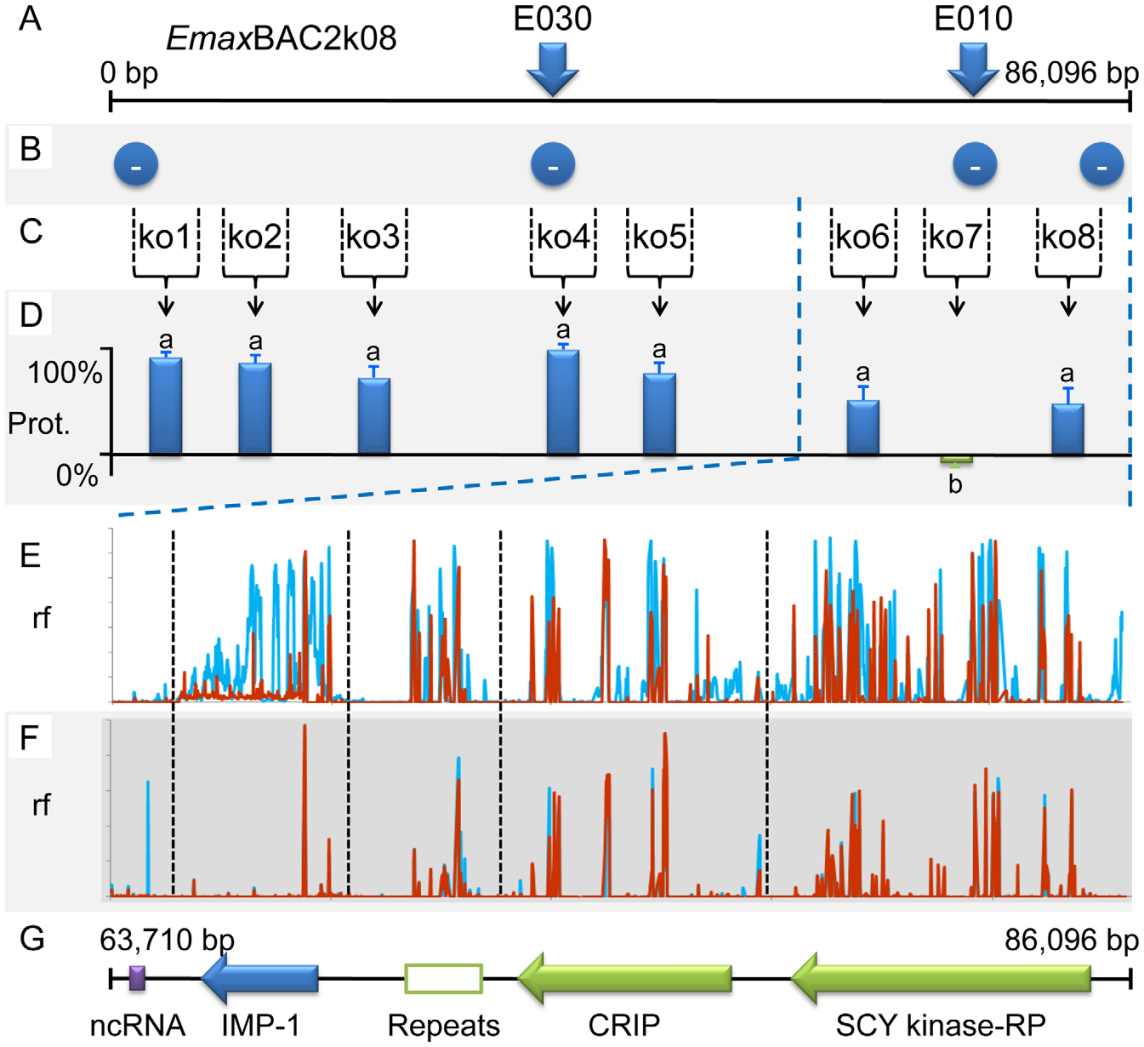
Fine mapping locus 5 correlated with susceptibility to strain-specific immune killing. (**A**) The distribution of strain-specific AFLP markers lost under immune selection across BAC *Emax*BAC2k08. (**B**) Recovery of strain-specific markers by immune selected backcross generations revealed that the entire BAC encoded-region remained absent in all generations (−). (**C**) Regions 1–8 selected for disruption by BAC recombineering. (**D**) The influence of targeted disruption on the ability of W strain-derived *Emax*BAC2k08 to confer cross-protective immunity when delivered by the heterologous *Eimeria maxima* H strain following transient transfection: the protective capacity retained by each recombineered BAC compared to the unmodified parent BAC. Bars marked with different letters were significantly different (p<0.05). (**E–F**) Relative fluorescence (rf) illustrating cDNA hybridisation to a BAC-tiling NimbleGen array covering ∼22.4 Kb of locus 5 corresponding to the smaller *Not* I/*Sfi* I BAC digest product. (**E**) cDNA derived from sporozoite (blue) and merozoite (67 hpi; red) lifecycle stages. (**F**) Profiles calculated from rf using cDNA derived from uninfected chicken intestinal tissue subtracted from chicken intestinal tissue 6 (blue) and 16 (red) hours post infection. (**G**) Transcribed sequences identified within locus 5 (repeats = a repetitive region, no evidence of transcription by targeted lifecycle stages, CRIP = putative cyclophilin-RNA interacting protein, RP = related protein.

**Figure 6:**
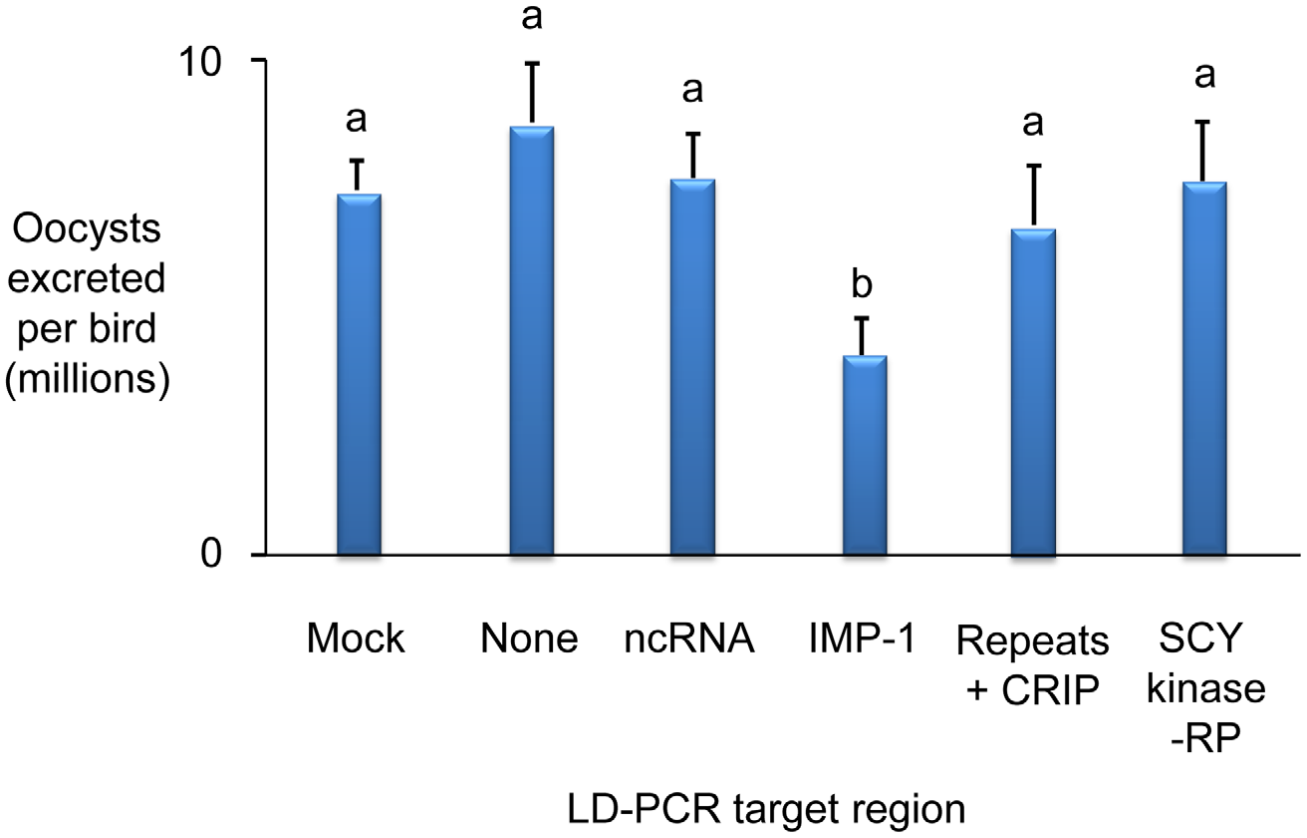
Immunising ability of individual genes identified within immune mapped locus 5. Average total *Eimeria maxima* W strain oocyst output per bird following challenge of birds immunised with *E. maxima* H vectored W strain transgenes identified within locus 5 presented as genomic LD-PCR amplicons. None = control, no immunisation, mock = control immunisation with no transgene, CRIP = putative cyclophilin-RNA interacting protein, RP = related protein. Bars marked with different letters were significantly different (p<0.05).

Whole BAC transient transfection identified locus 5, contained within BAC *Emax*BAC2k08, as capable of inducing the strongest protective immune response as judged by a reduction of oocyst production during challenge infection by 59.7% (**Table 1**). Backcross genotyping failed to provide any further focus, yielding a genomic region of interest spanning ∼90Kb (**Figure 5A–B**). The difference in locus size may reflect variation in genome-wide recombination rates, as have been reported for other Apicomplexa [15]. Two parallel approaches were used to focus the search for protective antigens, one based upon targeted disruption of regions predicted to contain open-reading frames and the second based upon defined fragments of the BAC purified from restriction digests. Targeted disruption by homologous recombination (BAC recombineering [16]; **Table S6**) at eight predicted coding regions (identified by similarity to other annotated genes or EST sequences and clusters of candidate open reading frames; **Figure 5C**) created a panel of eight otherwise unaltered *Emax*BAC2k08 versions (**Figure S1** and **S2**). The capacity of each daughter BAC to confer W-strain-specific protective immunity was tested by immunisation using the BAC transfection immunisation route followed by challenge with W strain parasites. For seven of the eight disrupted BACs no significant change in oocyst output was obtained compared with immunisation with the unmanipulated parent BAC. When region 7 was disrupted the protective effect was reduced to zero indicating that the important antigen or controlling element was associated with this region (**Figure 5D**). When the regions flanking region 7 (6 and 8) were disrupted a small, but non-significant reduction in oocyst output was observed, possibly resulting from disruption of associated controlling or stabilising sequences. In parallel we analysed purified BAC sub-sections that resulted from *Not* I/*Sfi* I digestion of *Emax*BAC2k08, which yielded ∼63.7 and ∼22.4 Kb fragments of the insert separate from the vector, the smaller fragment including the candidate disrupted region. Immunisation using H strain sporozoites transiently transfected with either BAC fragment confirmed the induction of a cross-protective immune phenotype associated with the smaller but not the larger fragment (40% and −16% protection respectively compared to a randomly selected BAC control). Similarity-led gene annotation suggested the presence of two coding sequences within the ∼22.4 Kb locus. More detailed scrutiny using a locus-wide NimbleGen tiling array with cDNA derived from *E. maxima* W strain sporozoites, merozoites (harvested 67 hours post infection, hpi) and chicken intestine centred on Meckel's diverticulum without infection or 6 and 16 hpi with 1×10^6^ W strain oocysts, revealed four sequences transcribed by the stages tested (**Figure 5E–G**). Although eimerian lifecycles are relatively complex [5], the choice of lifecycle stages to be sampled was informed by quantitative PCR-based enumeration of *E. maxima* replication in naive and immunised Line C chickens, which revealed the first 24 hpi to cover the period of most immune killing during homologous challenge (∼88% reduction at 20 hpi; **Figure S3**). Examination of the array-identified transcribed sequences revealed (i) a putative non-coding RNA, (ii) an unknown coding sequence, (iii) a putative cyclophilin-RNA interacting protein and (iv) a SCY kinase-related protein (FN813225–8). LD PCR amplicons covering each predicted W strain coding sequence and flanking regions, including a section of repeats not found to be transcribed but identified as a cross-reactive feature by the array, were used in the BAC transfection immunisation assay. Protective immunity against W strain challenge was only evident with the unknown coding sequence (**Figure 6**). To confirm the protective capacity of this antigen, which we now term ‘immune mapped protein-1’ (IMP-1), we produced and purified bacterially-expressed protein and vaccinated Line C chickens. *E. maxima* IMP-1 recombinant protein induced 45% immune protection against challenge by the W strain as judged by reduction in oocyst output (compared with the thioredoxin protein control, 50% compared with the unimmunised control; **Figure S4**). Comparison between the H and W IMP-1 coding sequences revealed five nucleotide polymorphisms, two of which yield non-synonymous changes in amino acid sequence.

Interrogation of the IMP-1 sequence using identification/prediction platforms including Phobius [17], SignalP [18], and SMART [19] suggest the absence of a classical signal peptide or recognisable domains. Nonetheless, predicted homologues can be identified within the genomes of other coccidial parasites including *E. tenella* (2e-87; FN813229; NCBI BLASTp2seq [20]), *Toxoplasma gondii* (XM_002370108; 2e-36) and *Neospora caninum* (GeneDB NCLIV_000430; 4e-37), all of which share a common intron/exon structure (**Figure S5**). Further work on the biology of IMP-1 and the eimerian AMA-1 may reveal characteristics common to molecules that are capable of inducing strong protective immunity.

Overall, our finding that just six regions of the genome were affected by strong immune selection is important since it suggests that protective immunity is focussed on a limited repertoire of parasite antigens. The genome of *Eimeria* spp. is estimated to be between 55 and 60 Mbp in size, encoding 8,000–9,000 genes (http://www.genedb.org/Homepage/Etenella), and the adaptive immune system recognises a large number of antigens. Our data indicate, in this precisely controlled genetic context, that natural infection-induced protective immunity is focussed on the recognition of only a small subset of the antigenic repertoire expressed by the parasite. This feature of antigenically complex pathogens has troubled those involved in vaccine development for many years. Our finding that strain-specific immunity against *E. maxima* is absolutely targeted against just six loci is comparable with the number identified with murine malaria [21] and highlights the concept that antigenically complex pathogens may only express small numbers of protective antigens. This feature of immunity may explain the lack of success in developing effective sub-unit vaccines against antigenically complex pathogens despite decades of effort directed at using immunodominant antigens. Indeed, although measurable responses will be directed against “protective antigens”, responses against “non-protective” or “poorly protective” antigens obscures effective antigen selection. Using response as the major selection criterion in antigen discovery pipelines confers high rates of false positive leads. Protection is a much more discriminatory tool that can be interrogated using a technically straightforward genetic mapping approach, focussing discovery on “protective antigens” and importantly supporting simultaneous consideration of all elements of the pathogen, identifying “sets of antigens” responsible for strong protective immunity. Understanding the basis for discrimination of antigenic molecules that stimulate ineffective responses from those that stimulate protective responses has the potential for impact far beyond the scope of this project.

In this report we document the application of a genetic approach to discover two protective antigens, one of which encodes an eimerian homologue of AMA-1 and the other a new vaccine candidate, IMP-1. The former raises an interesting possibility that there are features of certain molecules that confer sensitivity to protective immune responses across a wide range of Apicomplexa. Interestingly, homologues of the IMP-1 gene can be readily identified in non-eimerian apicomplexan parasites and these may also be candidate protective antigens. Three of the other four loci contain elements that confer strain specific protective immunity (by BAC transfection-immunisation studies) and it is likely that these also contain protective antigens. One alternative is that these regions may exert their effects indirectly (for example by regulation of other non-polymorphic loci) although at present this seems unlikely. The nature of the protective effects encoded by the remaining loci is the focus for ongoing studies. We propose that our strategy will contribute to development of new anti-eimerian vaccines and may have much broader impact on the development of vaccines against some of the most devastating parasitic diseases of humans and livestock.

## Materials and Methods

### Ethics statement

This study was carried out in strict accordance with the Animals (Scientific Procedures) Act 1986, an Act of Parliament of the United Kingdom. All animal studies and protocols were approved by the Institute for Animal Health Ethical Review Committee and the United Kingdom Government Home Office under the project licences 30/2047 and 30/2545.

### Parasites and animals

The *E. maxima* Houghton (H, sensitive to dietary robenidine) and Weybridge (W, resistant to 66 ppm dietary robenidine) strains were used as the parental parasites in these studies. Oocysts were propagated and genetic crosses were carried out *in vivo* as described previously [6].

### Experimental design: Genetic crosses and selection of progeny populations

Uncloned populations of hybrid parasites were derived from eight independent crosses between the H and W *E. maxima* strains in Line C White Leghorn chickens as described previously ([6]; oocysts recovered from between two and ten birds and pooled for each population). Hybrid sub-populations were derived from each cross following *in vivo* passage under a double selective barrier comprising W strain-specific immune (induced by previous infection with 100 oocysts of the pure W strain) and H strain-specific drug (66 ppm robenidine) selection [6]. All eight first generation selected parasite populations, together with four serially-selected populations (three to five rounds of selection, two to ten birds pooled per population per generation), were used to prepare the parasite mapping panel (**Table S2**).

One selected parasite population was backcrossed six times under double barrier selection, each backcross generation derived after *in vivo* phases of (i) cross fertilisation: *in vivo* passage using 100 sporulated hybrid oocysts with 400 sporulated W (immune-targeted) parental strain oocysts and (ii) selection: passage of 10,000 sporulated recovered parasites per bird under double barrier selection. Parasites recovered from ten birds were pooled for each backcross stage.

### Experimental design: *In vivo* protection trials

Experiments to measure immune protection induced by (i) immunisation through previous parasite exposure, (ii) recombinant protein or (iii) DNA vaccination followed standardised protocols. All treatment groups comprised six individually caged specific pathogen free Line C White Leghorn chickens. Total daily oocyst excretion per bird was determined following daily faecal collection from days 6–7, 7–8 and 8–9 post infection by flotation in saturated salt solution using a modified McMaster protocol as described previously [6]. All experiments included a non-immunised control group.

- Previous parasite exposure. Three week old chickens were immunised by oral infection with 100 sporulated *E. maxima* oocysts (wild type) or 1.0×10^6^ transfected *E. maxima* sporozoites (following oral gavage with 0.5 ml 5% w/v sodium bicarbonate solution; drug cleared three days post immunisation by inclusion of dietary robenidine at 66 ppm for three days). Six week old chickens were challenged with 250 sporulated *E. maxima* oocysts unless stated to be otherwise.
- Recombinant protein. Two week old chickens were immunised subcutaneously using 100 µg PBS-dialysed recombinant protein in TiterMax Gold adjuvant (Sigma-Aldrich Ltd.) at two sites in the neck. Chickens were subsequently re-immunised at three and four weeks of age (as before but in Freund's Incomplete adjuvant; Sigma-Aldrich Ltd.) prior to challenge with 250 sporulated *E. maxima* oocysts when six weeks old. Unimmunised, PBS alone and PBS-dialysed thioredoxin were included as negative control groups.
- DNA. Two week old chickens were immunised by intramuscular injection using 50 µg DNA vaccine plasmid in sterile phosphate buffered saline (pH 7.6) at two sites in the thigh. Chickens were subsequently re-immunised at three and four weeks of age (as before) in alternating legs prior to challenge with 250 sporulated *E. maxima* oocysts when six weeks old. TE alone and vector pcDNA3.1(+) (Invitrogen) were included as negative controls in addition to an unimmunised group. Tissue excised from the immunisation site post-mortem from one extra bird per group seven days post-final immunisation was processed to extract total RNA using the Qiagen RNeasy mini kit (Qiagen) for cDNA preparation and PCR to confirm DNA vaccine transcription (data not shown).

### Recombinant protein vaccine preparation

cDNA sequences corresponding to the predicted AMA-1 ectodomain and IMP-1 proteins (loci 1 and 5 respectively: AMA-1 coding nucleotides 79–1,347, IMP-1 161–1,275) were amplified from *E. maxima* W strain sporozoite cDNA, cloned into the expression vector pET32b (Novagen) using *Bam* HI/*Hind* III and *Nco* I/*Eco* RI restriction sites respectively and sub-cloned in *E. coli* BL21(DE3)pLysS (Novagen). Recombinant proteins were expressed and purified using HisTrap FF purification columns (GE Healthcare) as described by the manufacturer, dialysed overnight against PBS and finally mixed with an equal volume of adjuvant shortly before use. Thioredoxin expressed in the same manner using the unmodified pET32b vector was purified and used as a negative control.

### DNA vaccine preparation

cDNA sequences corresponding to the predicted AMA-1 ectodomain and sulphate transporter proteins (locus 1: AMA-1 coding nucleotides 79–1,347, sulphate transporter 1–2,988) were amplified from *E. maxima* W strain cDNA, cloned into the eukaryotic expression vector pcDNA3.1(+) (Invitrogen) using *Hind* III/*Bam* HI restriction sites and sub-cloned in *E. coli* XL1-Blue MRF (Stratagene). Plasmid DNA was purified using the Qiagen EndoFree Plasmid Maxi kit as recommended by the manufacturer (Qiagen), precipitated and re-suspended in endotoxin-free TE at 250 µg/ml.

### Nucleic acid resources

Genomic DNA was extracted from oocysts as described previously using a physical smashing step followed by phenol/chloroform extraction [22] and from chicken intestinal tissue samples using a Qiagen DNeasy tissue kit as described by the manufacturer (Qiagen) followed by RNase A treatment [23]. Total RNA was purified from *E. maxima* sporozoite, merozoite (harvested 67 hours post infection), infected and uninfected chicken intestinal tissue using a Qiagen RNeasy kit as described by the manufacturer [24]. A BAC library was constructed for the *E. maxima* W strain in the pBACe3.6 vector following the protocol of Osoegawa et al [25] based upon chromosomal DNA prepared from *E. maxima* sporozoites as described elsewhere [26]. BAC DNA was prepared using the Qiagen Large-Construct kit as described by the manufacturer (Qiagen).

Standard PCR amplification was completed using BIO-X-ACT Short DNA Polymerase (Bioline Ltd.). Each PCR reaction contained 5 ng template DNA, 20 pmol of relevant forward and reverse primers, 0.5 U *Taq* polymerase, 10 mM Tris–HCl, 1.5 mM MgCl2, 50 mM KCl and 0.2 mM dNTPs. Standard cycle parameters were 1×(5 min at 94°C), 30×(1 min at 94°C, 1 min at 54–58°C and 1–5 min at 72°C) and 1×(10 min at 72°C). For LD PCR BIO-X-ACT Long DNA Polymerase (Bioline Ltd.) was used as recommended by the manufacturer. Where required cDNA was prepared using Invitrogen Superscript II reverse transcriptase and oligo dT as described by the manufacturer (Invitrogen Ltd.). PCR fragments were cloned using pGEM-T Easy (Promega) in XL1-Blue *Escherichia coli* (Stratagene), miniprepped (Qiagen) and sequenced (Beckman CEQ 8000 genetic analysis system) as described by the respective manufacturers. Sequence assembly, annotation and interrogation were undertaken using VectorNTI v11.0 (Invitrogen) except where stated.

### Quantification of parasite replication

Groups of four inbred SPF Line C White Leghorn chickens were either left naive or were immunised by infection with 100 sporulated *E. maxima* W strain oocysts at three weeks of age. All birds were subsequently challenged by infection with 1.0×10^6^ *E. maxima* W strain oocysts at six weeks of age (homologous challenge). Unimmunised and immunised groups were culled at 0, 2, 4, 6, 8, 12, 16, 20, 24, 32, 40, 48 and 72 hours post challenge, when an 8 cm length of intestine centred on Meckel's diverticulum was recovered post-mortem from each test bird and homogenised in sterile TE using a Qiagen TissueRuptor (230 V, 50/60 Hz). Total genomic DNA was extracted from three 25 µl aliquots of each sample using a Qiagen AllPrep DNA/RNA Mini kit as described by the manufacturer (Qiagen). The total number of *E. maxima* genomes per host genome was determined from each sample using TaqMan quantitative PCR assays specific for the *E. maxima* microneme protein 1 (MIC1) and chicken glyceraldehyde 3-phosphate dehydrogenase (GAPDH) loci in duplex with the 7500 Fast Real-Time PCR System (Applied Biosystems) [23]. TaqMan probes were 5′ labeled with FAM (MIC1) or Yakima Yellow (GAPDH) and 3′ quenched with Eclipse Dark Quencher (Eurogentec). TaqMan conditions and cycle parameters were modified from the standard Applied Biosystems Fast protocol (1×95°C, 20 s; 40×95°C, 15 s and 60°C, 30 s). Quantitative calculations were facilitated and validated by comparison with known concentrations of the relevant genomic DNA template.

### Genetic marker production

AFLP was used to generate the majority of the genetic markers used during these studies as described elsewhere [27]. Approximately 50 ng total *E. maxima* genomic DNA was digested using one of five restriction enzyme combinations (**Table S1**; New England Biolabs) prior to ligation to adapters derived from those described by Vos et al, adapted for the respective restriction enzyme [27]. Primer pairs (MWG Biotech (UK) Ltd.) were based on the adaptor sequences and provided 0 and 1 (primary amplification) or 1 and 2 (secondary amplification) selective bases, respectively. Markers of interest were gel excised, re-amplified, cloned and sequenced as described previously [6]. Marker specific-primers were designed using Primer3 to amplify marker-associated DNA fragments [28].

Additional genetic markers were developed by sequencing 600–750 bp sections of *E. maxima* H strain genomic DNA corresponding to targets distributed across each locus mapped in the W strain (**Table S4** for the primers used, FN813230–41). Strain-specific primer pairs were developed following sequence alignment and SNP identification (ClustalX [29]; **Table S5**).

### Pulsed field gel electrophoresis

Chromosomal karyotypes were resolved by PFGE. Briefly, *E. maxima* W strain chromosomal DNA in ∼4 mm×3 mm×2 mm sections cut from an agarose block [26] was separated in a 0.8% SeaKem HGT agarose gel (Lonza) prepared in 0.5×Tris–borate EDTA (TBE) buffer and subjected to PFGE in a 21 cm×14 cm gel using a CHEF DRII system (Bio-Rad) in 2 L of 0.5×TBE running buffer at 14°C. PFGE conditions were (i) 216 h at 1.3 volts/cm with a switch time of 3,000–3,500 s followed by (ii) 120 h at 1.3 volts/cm with a switch time of 3,300–3,600 s finishing with (iii) 48 h at 1.2 volts/cm with a switch time of 3,200–3,400 s. Gels were stained in a 0.5 µg/ml aqueous solution of ethidium bromide for 30 min, destained in water and then photographed. *Hansenula wingei* and *Schizosaccharomyces pombe* DNA plugs (Bio-Rad) were included as size markers.

Individual BAC clone insert size was determined by *Not* I (New England Biolabs) digestion followed by PFGE in 1% Bio-Rad pulsed field certified agarose prepared in 0.5×TBE as above. PFGE conditions were 18 h at 4 volts/cm with a switch time of 1–6 s. Low range PFG size markers (New England Biolabs) were included as size markers.

### DNA hybridisation

PFGE-resolved chromosomal DNA was transferred to Hybond-N+ membrane (Amersham Biosciences) as recommended by the manufacturers. A total of 3072-BAC transformed *E. coli* DH10B were robotically gridded onto replicated filter arrays providing ∼7.5-fold coverage of the *E. maxima* W strain genome. PCR products derived from AFLP markers of interest were labelled with ^32^P using a Prime-It II random priming kit (Stratagene) and hybridised to filters for 16–24 h at 65°C as described by Amersham Biosciences. Filters were washed three times at 65°C in 0.1×SSC before exposure to X-ray film (Kodak BioMax MS) at −80°C against intensifying screens.

### BAC sequencing, assembly and annotation

BAC DNA was prepared from clones identified by hybridisation to AFLP markers of interest using the Qiagen Large-Construct kit as recommended by the manufacturer. Small insert libraries (2–4 Kb) were prepared by shearing the DNA by sonication, blunt ending and size selecting by agarose gel electrophoresis prior to sub-cloning into *Sma* I digested dephosphorylated pUC18 for use in a whole-BAC shotgun sequencing strategy. ABI PRISM BigDye Terminator (Applied Biosystems) forward and reverse plasmid end sequences generated using an ABI3730 capillary sequencer were assembled using the Staden-based PHRAP (P. Green, unpublished). Contig assembly was based upon LD PCR.

Preliminary annotation of each assembled BAC sequence was achieved using tBLASTx [20] interrogation of all publically accessible sequences through the National Center for Biotechnology Information (NCBI, http://www.ncbi.nlm.nih.gov/), supplemented by *Eimeria* species EST data from the *Eimeria* ORESTES and *E. maxima* EST sequencing projects (Gruber and Madeira, unpublished; Wan and Blake, unpublished, respectively). All sequences produced in this study have been submitted to EMBL, where they are available under the accession numbers FN813211–44.

### BAC recombineering

The *E. coli* DY380 strain [16] (kindly supplied by the National Cancer Institute, Frederick, USA) was initially transformed with *E. maxima* W strain BAC *Emax*BAC2K8 as described elsewhere [30]. Subsequently, the DY380/*Emax*BAC2K8 line was transformed with each of eight PCR products representing (i) unique *Emax*BAC2K8 sequences for targeted homologous recombination and (ii) the β-lactamase coding sequence amplified from pGEM-T Easy (Promega; primers as shown in **Table S6**; PCR as above, purified using the Qiagen Gel Extraction kit as described by the manufacturer) [30]. The resulting bacterial strains were sub-cloned and tested for evidence of correctly targeted insertion in a pure clonal line and the absence of widespread BAC disruption by (i) positive PCR between insert and flanking BAC sequences (**Figure S1**), (ii) negative PCR between target and flanking BAC sequences (**Figure S1**, primers shown in **Table S7**) and (iii) unchanged BAC PFGE profile following *Not* I/*Sfi* I digestion (**Figure S2**; enzymes New England Biolabs).

### Transfection

*E. maxima* W strain genomic DNA, presented as whole, recombineered or partial BAC-encoded templates or LD PCR amplicons, was used to transiently transfect the *E. maxima* H strain. For whole and recombineered BAC transfection purified plasmid DNA was re-suspended at 50 µg/10 µl TE. BAC *Emax*BAC2k08 was subdivided by *Not* I/*Sfi* I digestion (New England Biolabs) and subsequent PFGE (as above), yielding ∼63.7 and ∼22.4 Kb fragments of the insert as well as the vector. The 63.7 and 22.4 Kb fragments were gel excised following large-scale PFGE, electroeluted into dialysis bags in TE, precipitated and re-suspended at 37 and 13 µg/10 µl respectively in TE as described elsewhere [31]. LD PCR amplicons representing three *Emax*BAC8f18 candidate regions and four *Emax*BAC2k08 regions were amplified in triplicate, (primers shown in **Table S8**, designed to lie>1 Kb outside any BLAST hit with an E value of 1e-05 or below or to yield an amplicon>7 Kb in size across the predicted coding sequence, whichever was the greater). All triplicates were electrophoresed to check for purity and target size, identified by test secondary PCR (**Table S8**), pooled once validated, precipitated and re-suspended in 10 µl TE [31].

Transient transfection was accomplished following a protocol modified slightly from that described previously [10]. Briefly, freshly excysted and purified *E. maxima* H strain sporozoites were washed in incomplete cytomix and re-suspended in AMAXA Basic Parasite Nucleofector Solution 2 at 3.0×10^7^/ml immediately prior to nucleofection. Subsequently, 100 µl sporozoite suspension was mixed with 10 µl DNA at room temperature, transferred to a cuvette and nucleofected as described by the manufacturer using a Nucleofector II with program U-033 (Lonza). Post-nucleofection all sporozoites were immediately re-suspended in 3 ml PBS+1% glucose (w/v) and left to rest for 20 mins at room temperature. Sporozoites were then either used as an oral dose to initiate *in vivo* infection or incubated overnight at 41°C in a 5% CO_2_ incubator. For oral dosing the output from two nucleofections were pooled and dosed using 1.0×10^6^ sporozoites (counted pre-nucleofection) per bird. Total RNA was extracted from incubated sporozoites using the Qiagen RNeasy mini kit for cDNA preparation and PCR of one or more transfected DNA-specific transgenes to confirm transfection (data not shown).

### BAC tiling array

A custom-made NimbleGen genome tiling array containing 79,955 50–75 bp probes (presented as forward and reverse strands in duplicate) was designed and produced using BAC sequences obtained during these studies (Roche). *E. maxima* W strain genomic DNA covered by BACs *Emax*BAC8f18 and *Emax*BAC2k08 were represented by 9,798 and 7,387 probes respectively (*Emax*BAC8f18: 8,321 unique, 1,477 twice, 84.5% coverage; *Emax*BAC2k08: 7,315 unique, 72 twice, 95.5% coverage). Total RNA extracted from purified *E. maxima* W strain sporozoites, merozoites (harvested 67 hpi), uninfected chicken intestinal tissue sampled at Meckel's diverticulum and chicken intestinal tissue sampled 6 and 16 hpi with 1×10^6^ *E. maxima* W strain oocysts were processed to produce labelled cDNA and hybridised to the array as described by the manufacturer (Roche). Arrays were scanned using a GenePix 4000B scanner and the images were visualised using GenePix Pro software (Axon). Array design was viewed using NimbleGen SignalMap v1.9 software. Arrays were read using NimbleGen NimbleScan v2.5 software with alignment and uniformity cut off points of 0.15 and 0.3 respectively.

### Statistics

Arithmetic mean and associated standard error of the mean (SEM) for each sample or group were calculated using Excel (Microsoft Excel 2002, Microsoft Corporation, 2001). Statistical analyses were performed using the t-test, ANOVA, Chi^2^ or Kruskal-Wallis tests in Minitab (Minitab Release 14, Minitab Inc., 2003), complimented by post hoc analysis using the Tukey's test. Differences were deemed significant with a *p* value<0.05.

### Accession numbers[Table ppat-1001279-t002]

**Table ppat-1001279-t002:** 

Sequence ID	Acc. No.
*Eimeria maxima* AFLP fragments	FN813211–FN813218
*Eimeria maxima* partial mRNA for putative sulphate transporter, strain Weybridge	FN813219
*Eimeria maxima* partial mRNA for putative sulphate transporter, strain Houghton	FN813220
*Eimeria maxima* mRNA for putative apical membrane antigen-1 (ama-1 gene), strain Weybridge	FN813221
*Eimeria maxima* mRNA for putative apical membrane antigen-1 (ama-1 gene), strain Houghton	FN813222
*Eimeria maxima* partial mRNA for putative transcription elongation factor (tef gene), strain Weybridge	FN813223
*Eimeria maxima* partial mRNA for putative transcription elongation factor (tef gene), strain Houghton	FN813224
*Eimeria maxima* EST, strain Weybridge, clone EmaxBAC2k08_NIM1_W	FN813225
*Eimeria maxima* EST, strain Houghton, clone EmaxBAC2k08_NIM1_H	FN813226
*Eimeria maxima* mRNA for IMP1 protein, strain Weybridge	FN813227
*Eimeria maxima* mRNA for IMP1 protein, strain Houghton	FN813228
*Eimeria tenella* mRNA for IMP1 protein, strain Houghton	FN813229
*Eimeria maxima* SCAR fragments	FN813230–FN813241
*Eimeria maxima* BAC clone EmaxBAC7a02	FN813242
*Eimeria maxima* BAC clone EmaxBAC8f18	FN813243
*Eimeria maxima* BAC clone EmaxBAC2k08	FN813244

## Supporting Information

Figure S1 PCR confirmation of targeted EmaxBAC2k08 disruption by recombineering for eight selected regions (1–8). PCR assays confirming the targeted insertion of the recombineering cassette (knockout: k/o) and the absence of unmodified BAC copies (wild type: wt). + = recombineered candidate BAC, − = unmodified original BAC.
(0.30 MB TIF)

Figure S2 Demonstration of the absence of gross BAC conformational change following recombineering. PFGE resolution of *Not I/Sfi* I digested unmodified *Emax*BAC2k08 (lane 1) and *Emax*BAC2k08 recombineered to disrupt regions 1–8 (lanes 2–9).
(1.71 MB TIF)

Figure S3 The influence of host immunity on intracellular *Eimeria maxima* W strain replication revealed by qPCR. *In vivo* intracellular *E. maxima* W strain replication in naïve (red, solid line) and previously infected (homologous infection; blue, broken line) chickens. Total parasite genome numbers determined for an 8 cm intestinal section centred upon Meckel's diverticulum. Region A highlights the first significant difference in parasite replication between the immunised and unimmunised host. Importantly, whilst qPCR-detected parasite genome numbers are not equivalent to viable parasites, the residual parasite genomes detected after 20 hpi in the immune host were not seen to replicate and were unlikely to represent live parasites. Thus, regions A and B represent the period when immune killing must have occurred. *p<0.01, **p<0.005.
(0.22 MB TIF)

Figure S4 Anticoccidial protective capacity of *Eimeria maxima* IMP-1. Anticoccidial protection induced by vaccination with recombinant *E. maxima* IMP-1 compared with thioredoxin, PBS and no immunisations (protein, immunisation and environmental controls respectively). Bars marked with different letters were significantly different (p<0.01, ANOVA + Tukey's post hoc).
(0.14 MB TIF)

Figure S5 Putative apicomplexan *IMP-1* homologues. (a) IMP-1 intron/exon structure from *Eimeria maxima* (FN813225), *Eimeria tenella* (FN813229), *Toxoplasma gondii* (XM_002370108) and *Neospora caninum* (NCLIV_000430; GeneDB). (b) Full length IMP-1 alignment using sequenced (*E. maxima*) or predicted (others) translated coding sequences.
(1.15 MB TIF)

Table S1 Frequency of five AFLP marker inheritance patterns. H parent specific, negative selection = 66 ppm dietary robenidine. W parent specific, negative selection = W strain-specific immunity induced by previous W strain infection. No significant difference was noted in the number of strain-specific markers amplified from either parental strain (Chi^2^ test). No significant bias was detected in the identification of negatively selected markers between the enzyme combinations (Kruskal-Wallis test).
(0.03 MB DOC)

Table S2 The *Eimeria maxima* mapping panel. nr = not relevant.
(0.03 MB DOC)

Table S3 Transient *Eimeria maxima* BAC transfection: Utility for in vivo immunisation and phenotypic screening for associated immunoprotective capacity. Immunisation included infection followed by drug clearance three days later using dietary robenidine (66 ppm). Challenge doses were administered three weeks after the immunising dose. Figures within each trial annotated with a different superscript letter were significantly different (p<0.05; ANOVA + Tukey's post hoc). *Control BAC = *Emax*BAC4c21, **Test BAC = *Emax*BAC2k08, mapped to locus 5. NF = none found.
(0.04 MB DOC)

Table S4 Primers for PCR amplification of equivalent *Eimeria maxima* H strain regions to identify polymorphic markers across locus 1 (BAC *Emax*BAC8f18).
(0.03 MB DOC)

Table S5 W strain-specific primers for genotyping backcrossed populations (derived using sequences from Table S4). Upper case letters are polymorphic between the *Eimeria maxima* H and W strains.
(0.04 MB DOC)

Table S6 BAC recombineering strategy. BAC recombineering constructs created by PCR amplifying a selectable cassette (β-lactamase, using pGEM-T Easy as template, primer sequences A) incorporating unique BAC sequences for targeted recombination (sequences B).
(0.06 MB DOC)

Table S7 Validation of targeted BAC recombineering by PCR. Primers designed to confirm targeted disruption: knockout = amplification between target-flanking genomic DNA and the insert, wild-type = amplification between target-flanking genomic DNA and the genomic DNA target. Results shown in Figure S4.
(0.04 MB DOC)

Table S8 *Emax*BAC8f18 and *Emax*BAC2k08 specific primers for LD PCR and nested PCR to confirm LD PCR amplicon identity. *Putative annotation.
(0.03 MB DOC)
