# Supplementary figures and images for: Genetic Mapping Identifies Novel Highly Protective Antigens for an Apicomplexan Parasite
Source: PLoS Pathog. 2011 Feb 10;7(2):e1001279. doi: 10.1371/journal.ppat.1001279 (PMC3037358; doi:10.1371/journal.ppat.1001279)

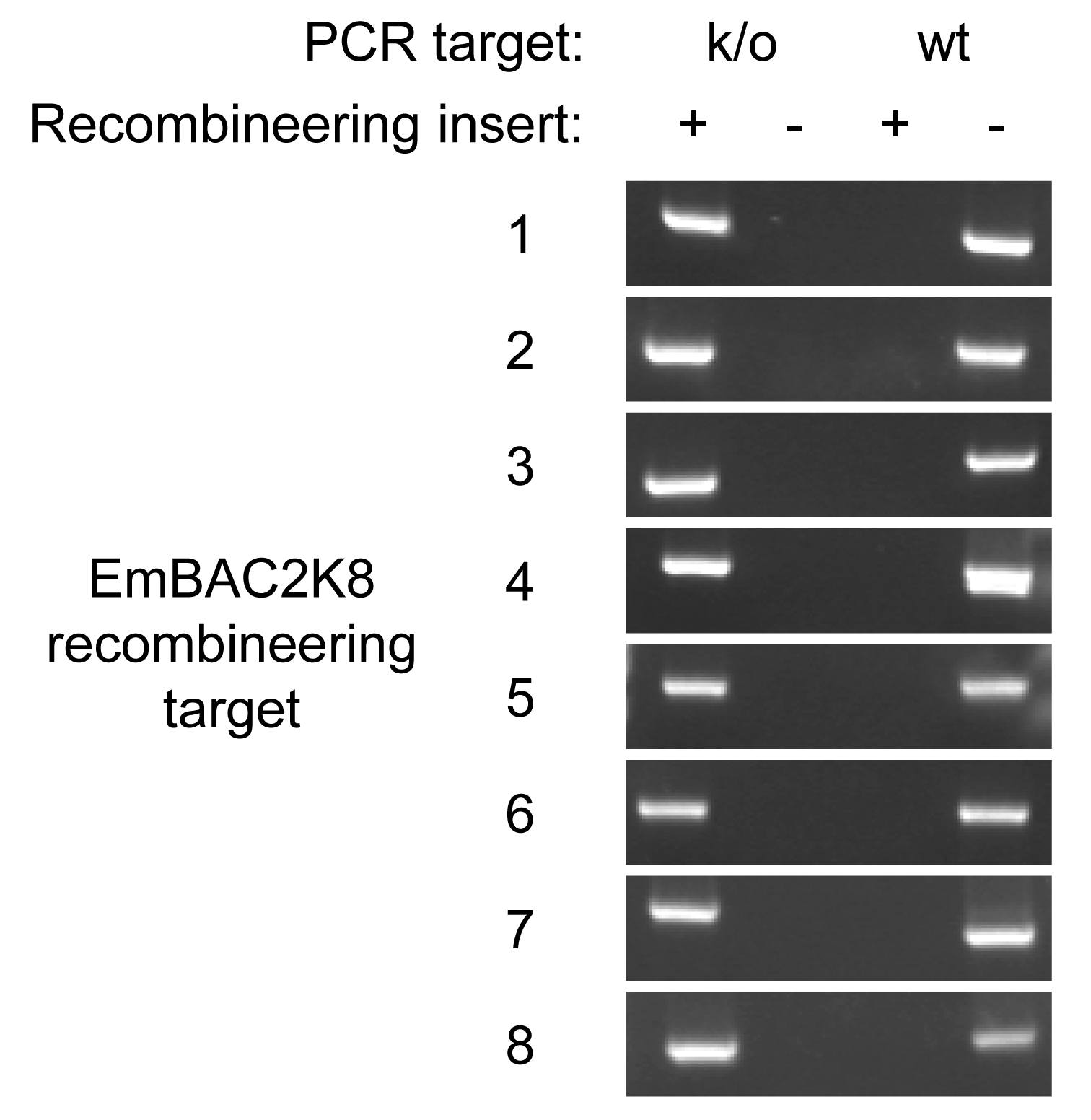

Supplement: Figure S1 — PCR confirmation of targeted EmaxBAC2k08 disruption by recombineering for eight selected regions (1–8). PCR assays confirming the targeted insertion of the recombineering cassette (knockout: k/o) and the absence of unmodified BAC copies (wild type: wt). + = recombineered candidate BAC, − = unmodified original BAC. (0.30 MB TIF) [file ppat.1001279.s001.tif]

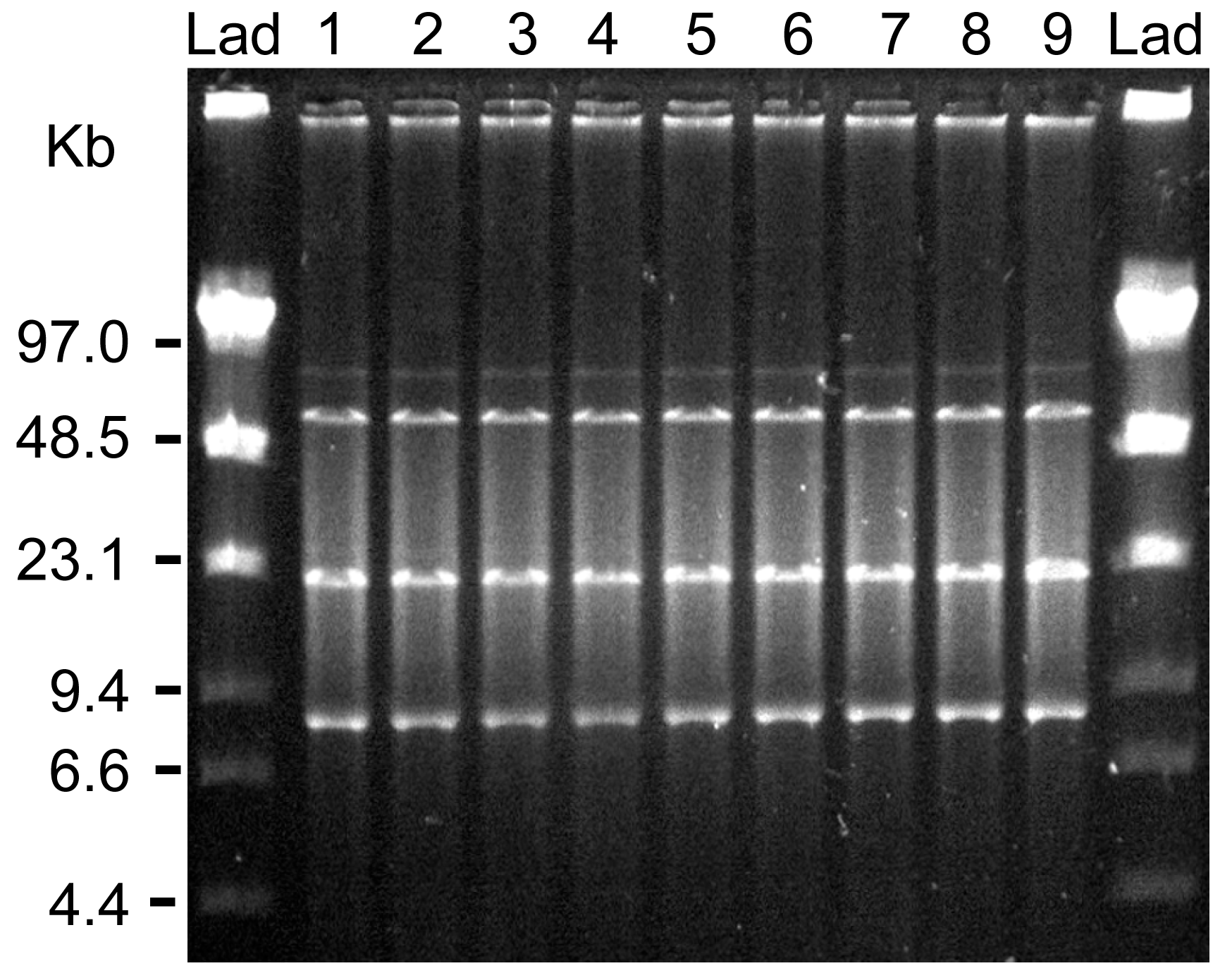

Supplement: Figure S2 — Demonstration of the absence of gross BAC conformational change following recombineering. PFGE resolution of Not I/Sfi I digested unmodified EmaxBAC2k08 (lane 1) and EmaxBAC2k08 recombineered to disrupt regions 1–8 (lanes 2–9). (1.71 MB TIF) [file ppat.1001279.s002.tif]

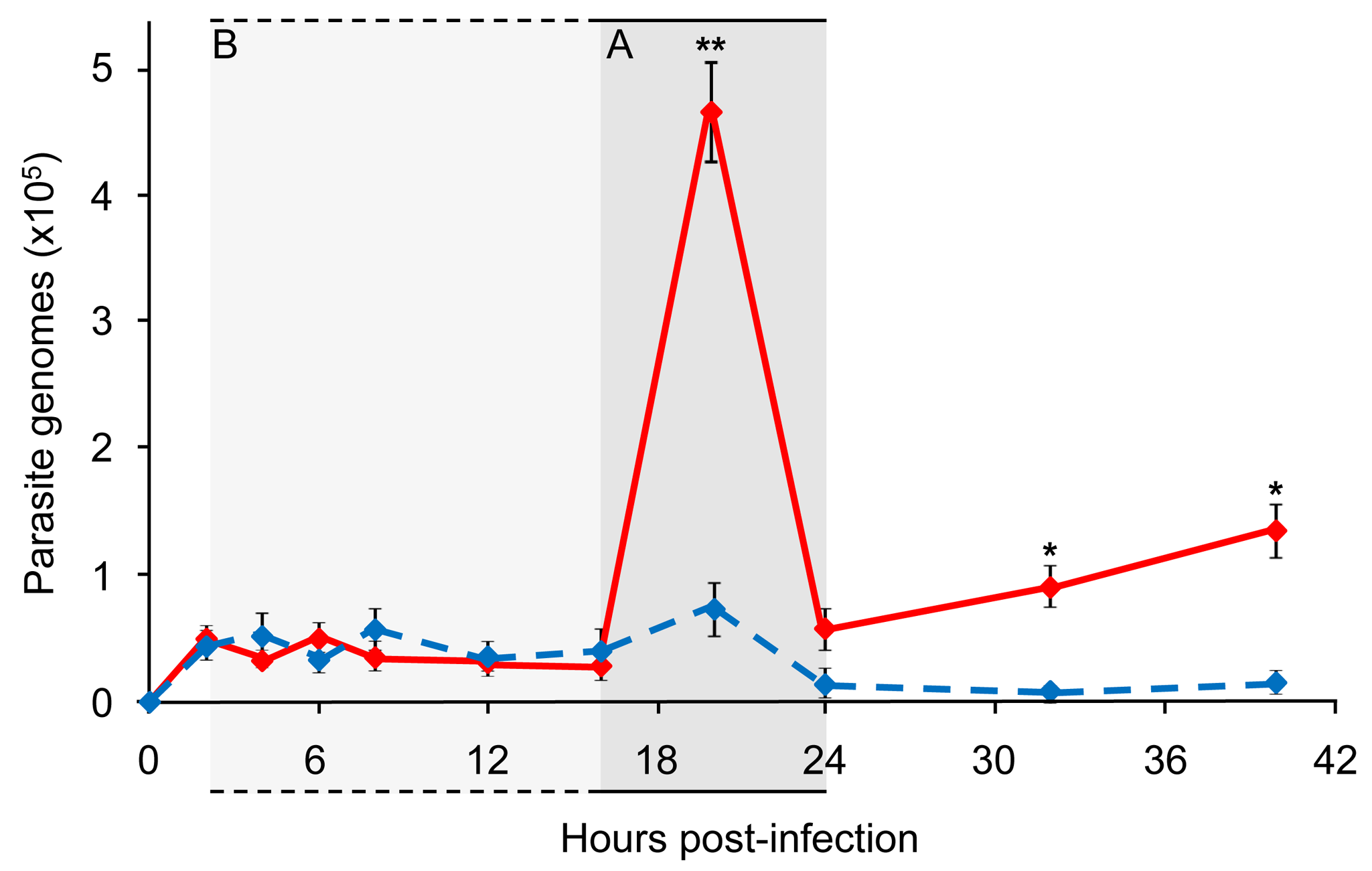

Supplement: Figure S3 — The influence of host immunity on intracellular Eimeria maxima W strain replication revealed by qPCR. In vivo intracellular E. maxima W strain replication in naïve (red, solid line) and previously infected (homologous infection; blue, broken line) chickens. Total parasite genome numbers determined for an 8 cm intestinal section centred upon Meckel's diverticulum. Region A highlights the first significant difference in parasite replication between the immunised and unimmunised host. Importantly, whilst qPCR-detected parasite genome numbers are not equivalent to viable parasites, the residual parasite genomes detected after 20 hpi in the immune host were not seen to replicate and were unlikely to represent live parasites. Thus, regions A and B represent the period when immune killing must have occurred. *p<0.01, **p<0.005. (0.22 MB TIF) [file ppat.1001279.s003.tif]

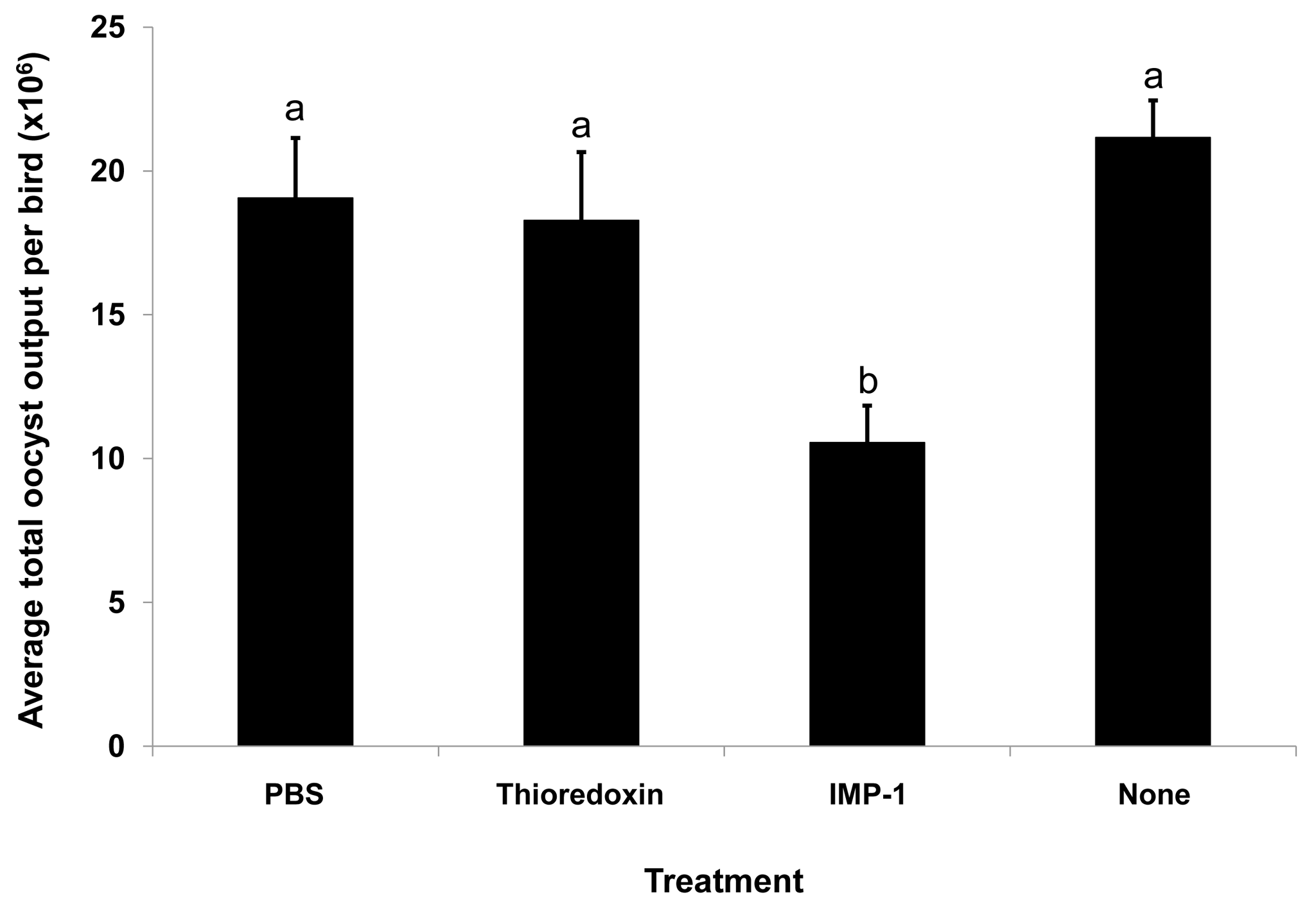

Supplement: Figure S4 — Anticoccidial protective capacity of Eimeria maxima IMP-1. Anticoccidial protection induced by vaccination with recombinant E. maxima IMP-1 compared with thioredoxin, PBS and no immunisations (protein, immunisation and environmental controls respectively). Bars marked with different letters were significantly different (p<0.01, ANOVA + Tukey's post hoc). (0.14 MB TIF) [file ppat.1001279.s004.tif]

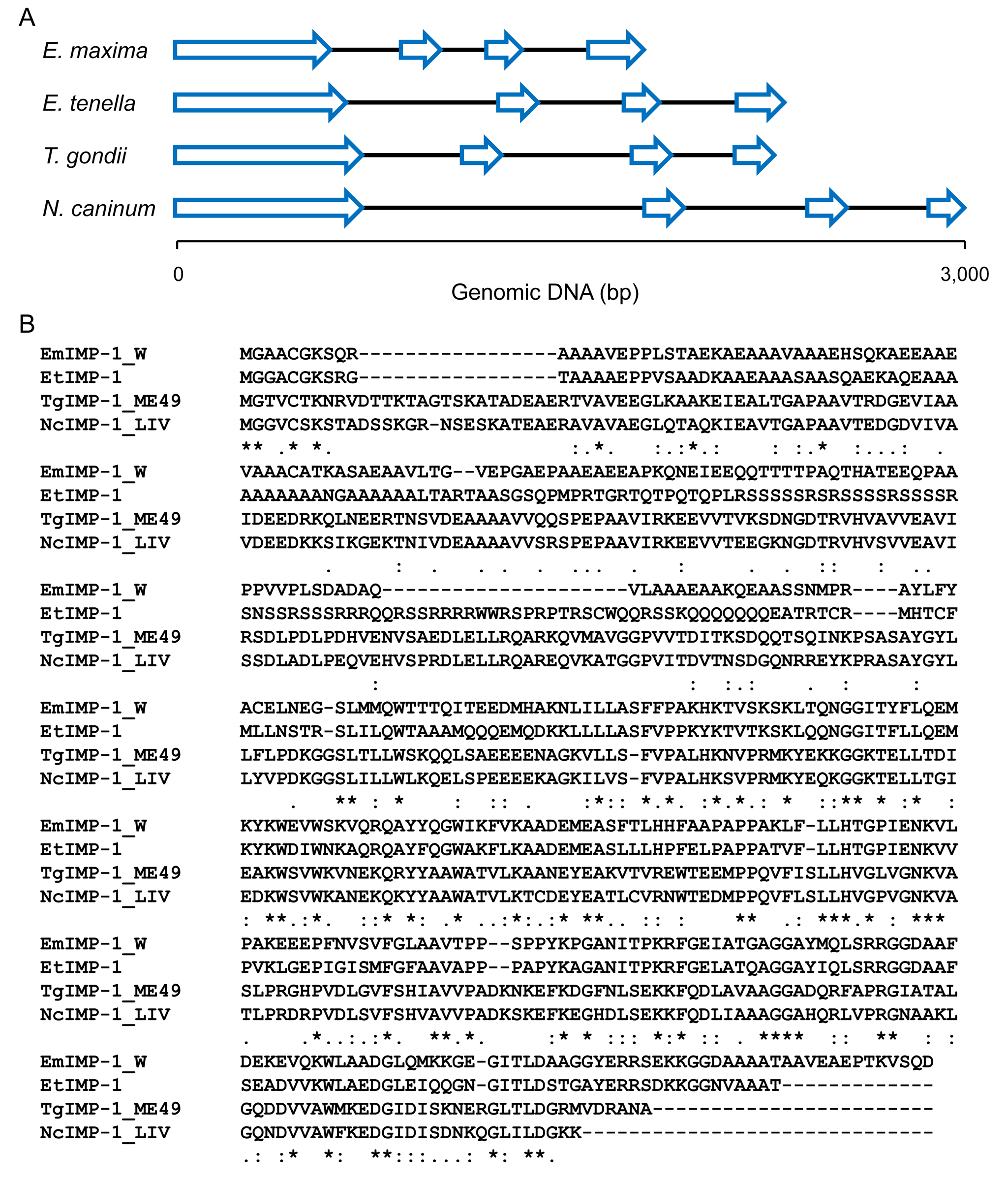

Supplement: Figure S5 — Putative apicomplexan IMP-1 homologues. (a) IMP-1 intron/exon structure from Eimeria maxima (FN813225), Eimeria tenella (FN813229), Toxoplasma gondii (XM_002370108) and Neospora caninum (NCLIV_000430; GeneDB). (b) Full length IMP-1 alignment using sequenced (E. maxima) or predicted (others) translated coding sequences. (1.15 MB TIF) [file ppat.1001279.s005.tif]
