# Supplementary material for: Genetic Mapping Identifies Novel Highly Protective Antigens for an Apicomplexan Parasite
Source: PLoS Pathog. 2011 Feb 10;7(2):e1001279. doi: 10.1371/journal.ppat.1001279 (PMC3037358; doi:10.1371/journal.ppat.1001279)
Supplement: Table S1 — Frequency of five AFLP marker inheritance patterns. H parent specific, negative selection = 66 ppm dietary robenidine. W parent specific, negative selection = W strain-specific immunity induced by previous W strain infection. No significant difference was noted in the number of strain-specific markers amplified from either parental strain (Chi2 test). No significant bias was detected in the identification of negatively selected markers between the enzyme combinations (Kruskal-Wallis test). (0.03 MB DOC) [file ppat.1001279.s006.doc]

**Table S1.** Frequency of five AFLP marker inheritance patterns.

|  | AFLP enzyme combinations | | | | |  |  |
| --- | --- | --- | --- | --- | --- | --- | --- |
| Pattern | *Eco* RI/*Mse* I | *Taq* I/*Pst* I | *Bgl* II/*Mse* I | *Bgl* II/*Pst* I | *Eco* RI/*Taq* I | Total | % total |
| Common | 890 | 110 | 96 | 904 | 108 | 2108 | 65.3 |
| H parent specific, unselected | 371 | 16 | 24 | 164 | 11 | 586 | 18.1 |
| W parent specific, unselected | 289 | 13 | 22 | 155 | 19 | 498 | 15.4 |
| H parent specific, negatively selected | 2 | 0 | 0 | 0 | 0 | 2 | 0.1 |
| W parent specific, negatively selected | 19 | 0 | 0 | 15 | 2 | 36 | 1.1 |
| Total | 1550 | 139 | 142 | 1223 | 138 | 3230 | 100.00 |

H parent specific, negative selection = 66 ppm dietary robenidine. W parent specific, negative selection = W strain-specific immunity induced by previous W strain infection. No significant difference was noted in the number of strain-specific markers amplified from either parental strain (Chi^2^ test). No significant bias was detected in the identification of negatively selected markers between the enzyme combinations (Kruskal-Wallis test).
