# Supplementary material for: Genetic Mapping Identifies Novel Highly Protective Antigens for an Apicomplexan Parasite
Source: PLoS Pathog. 2011 Feb 10;7(2):e1001279. doi: 10.1371/journal.ppat.1001279 (PMC3037358; doi:10.1371/journal.ppat.1001279)
Supplement: Table S2 — The Eimeria maxima mapping panel. nr = not relevant. (0.03 MB DOC) [file ppat.1001279.s007.doc]

**Table S2.** The *Eimeria maxima* mapping panel.

| Parasite population | Selection during passage | Generation(s) | Number of populations |
| --- | --- | --- | --- |
| H strain (parent) | None | nr | 1 |
| W strain (parent) | None | nr | 1 |
| Progeny of an H/W strain cross | None | 1 | 8 |
| Progeny of an H/W strain cross | W strain-specific immunity/robenidine | 1 | 8 |
| Progeny of an H/W strain cross | None | 2-3 | 4 |
| Progeny of an H/W strain cross | W strain-specific immunity/robenidine | 3-5 | 4 |
| Backcrossed progeny of an H/W strain cross | W strain-specific immunity/robenidine | 1-6 | 1 |

nr = not relevant.
