# Supplementary material for: Genetic Mapping Identifies Novel Highly Protective Antigens for an Apicomplexan Parasite
Source: PLoS Pathog. 2011 Feb 10;7(2):e1001279. doi: 10.1371/journal.ppat.1001279 (PMC3037358; doi:10.1371/journal.ppat.1001279)
Supplement: Table S3 — Transient Eimeria maxima BAC transfection: Utility for in vivo immunisation and phenotypic screening for associated immunoprotective capacity. Immunisation included infection followed by drug clearance three days later using dietary robenidine (66 ppm). Challenge doses were administered three weeks after the immunising dose. Figures within each trial annotated with a different superscript letter were significantly different (p<0.05; ANOVA + Tukey's post hoc). *Control BAC = EmaxBAC4c21, **Test BAC = EmaxBAC2k08, mapped to locus 5. NF = none found. (0.04 MB DOC) [file ppat.1001279.s008.doc]

**Table S3.** Transient *Eimeria maxima* BAC transfection: utility for *in vivo* immunisation and phenotypic screening for associated immunoprotective capacity.

| Trial | Immunisation | | Electroporation | Challenge | Oocysts excreted per bird |
| --- | --- | --- | --- | --- | --- |
|  | Vehicle | BAC |  |  | ± SEM (x10^6^) |
| 1 | None | None | No | *E. maxima* H | 19.4 ± 1.8^a^ |
|  | *E. maxima* H | None | No | *E. maxima* H | NF |
|  |  |  |  |  |  |
| 2 | None | None | No | *E. maxima* W | 25.8 ± 2.3^a^ |
|  | *E. maxima* H | None | Yes | *E. maxima* W | 22.0 ± 2.6^a^ |
|  | *E. maxima* H | Control* | Yes | *E. maxima* W | 24.4 ± 2.7^a^ |
|  | None | Test** | Yes | *E. maxima* W | 24.6 ± 2.0^a^ |
|  | *E. maxima* H | Test** | No | *E. maxima* W | 21.0 ± 3.9^a^ |
|  | *E. maxima* H | Test** | Yes | *E. maxima* W | 8.1 ± 0.7^b^ |

Immunisation included infection followed by drug clearance three days later using dietary robenidine (66 ppm). Challenge doses were administered three weeks after the immunising dose. Figures within each trial annotated with a different superscript letter were significantly different (p<0.05; ANOVA + Tukey’s post hoc). *Control BAC = *Emax*BAC4c21, **Test BAC = *Emax*BAC2k08, mapped to locus 5. NF = none found.
