# Supplementary material for: Genetic Mapping Identifies Novel Highly Protective Antigens for an Apicomplexan Parasite
Source: PLoS Pathog. 2011 Feb 10;7(2):e1001279. doi: 10.1371/journal.ppat.1001279 (PMC3037358; doi:10.1371/journal.ppat.1001279)
Supplement: Table S4 — Primers for PCR amplification of equivalent Eimeria maxima H strain regions to identify polymorphic markers across locus 1 (BAC EmaxBAC8f18). (0.03 MB DOC) [file ppat.1001279.s009.doc]

**Table S4.** Primers for PCR amplification of equivalent *Eimeria maxima* H strain regions to identify polymorphic markers across locus 1 (BAC *Emax*BAC8f18).

| Genetic marker | Forward primer | Reverse primer |
| --- | --- | --- |
| EmBAC8f18_01w | 5’-ttgagttgggcgattttccttt-3’ | 5’-acgaggacacgtagctggtctg-3’ |
| EmBAC8f18_02w | 5’-cgcgtgtgtggtacaaacaaaa-3’ | 5’-agtccatggcactccacacaat-3’ |
| EmBAC8f18_03w | 5’-taccgagcaacaaggcaggtaa-3’ | 5’-tcccatattttgccatttgctg-3’ |
| EmBAC8f18_04w | 5’-ctggtggacgatgttctggttc-3’ | 5’-gtctattcaacaccggcagtcg-3’ |
| EmBAC8f18_05w | 5’-ggatcacattccgttcaactcg-3’ | 5’-tcgacagctgcaaaagaaaagc-3’ |
| EmBAC8f18_06w | 5’-cttctgatctcccgctttcaca-3’ | 5’-tcttgccgttgttctcgtcttc-3’ |
| EmBAC8f18_07w | 5’-ttccctttttgcgaatctccat-3’ | 5’-cctctggctgaaagaagcaaca-3’ |
| EmBAC8f18_08w | 5’-gtcaggccaataacagccacag-3’ | 5’-ccccatccatccttagaagtgc-3’ |
| EmBAC8f18_09w | 5’-tttcctccttttccccctcttc-3’ | 5’-accgaaggaaaagggtttaggg-3’ |
| EmBAC8f18_10w | 5’-cctccctccctctttttccttg-3’ | 5’-agcagcagcaggaagagtgtgt-3’ |
| EmBAC8f18_11w | 5’-gggagaggaaggggagactgat-3’ | 5’-tcctcgattctacctgctgctg-3’ |
| EmBAC8f18_12w | 5’-agcaagcaatgacaagcacctc-3’ | 5’-gtggatgggtggggattatagg-3’ |
