# Supplementary material for: Genetic Mapping Identifies Novel Highly Protective Antigens for an Apicomplexan Parasite
Source: PLoS Pathog. 2011 Feb 10;7(2):e1001279. doi: 10.1371/journal.ppat.1001279 (PMC3037358; doi:10.1371/journal.ppat.1001279)
Supplement: Table S5 — W strain-specific primers for genotyping backcrossed populations (derived using sequences from Table S4). Upper case letters are polymorphic between the Eimeria maxima H and W strains. (0.04 MB DOC) [file ppat.1001279.s010.doc]

**Table S5.** W strain-specific primers for genotyping backcrossed populations (derived using sequences from Table S4).

| Genetic marker | Forward primer | Reverse primer |
| --- | --- | --- |
| EmBAC8f18_01w | 5’-tgagacgggaactttgcttctG-3’ | 5’-tagcatcgagaaatcgccgttA-3’ |
| EmBAC8f18_02w | 5’-cttggctcaccaatgtcacctC-3’ | 5’-accttcctttaccattccgacT-3’ |
| EmBAC8f18_03w | 5’-agtctcctttggtacccggtgA-3’ | 5’-cttgcgtatccatgatagctcT-3’ |
| EmBAC8f18_04w | 5’-gtgggtaagcgctttggagagT-3’ | 5’-tgggctctacagcagctgaaaC-3’ |
| EmBAC8f18_05w | 5’-tgtcatcgacaaggaacatctcT-3’ | 5’-gtgcttctttcggaggcatctT-3’ |
| EmBAC8f18_06w | 5’-ttctgaaatggggtgtatagacG-3’ | 5’-ctaaaacctcttccctgggC-3’ |
| EmBAC8f18_07w | 5’-aaaagattagttgaatatctgaggaGA-3’ | 5’-gaagcaacaagcgcatctaatC-3’ |
| EmBAC8f18_08w | 5’-agagggaaaatcaatgcaagaC-3’ | 5’-gcagcagcaGCTgctgCT-3’ |
| EmBAC8f18_09w | 5’-gtgtgcgtgataaggagccC-3’ | 5’-atctaaaagtaagtcttttcttcttttttTC-3’ |
| EmBAC8f18_10w | 5’-gcttactctgtatagtaccatccttattT-3’ | 5’-ttattttttctcctgctGctC-3’ |
| EmBAC8f18_11w | 5’-gatggatccgtaggtggtgttG-3’ | 5’-tgctgcagtgtctgtttgcttC-3’ |
| EmBAC8f18_12w | 5’-ctcagggcagTctagtcttcC-3’ | 5’-gattgcattcagtcgtgacagC-3’ |

Upper case letters are polymorphic between the *Eimeria maxima* H and W strains.
