# Supplementary material for: Genetic Mapping Identifies Novel Highly Protective Antigens for an Apicomplexan Parasite
Source: PLoS Pathog. 2011 Feb 10;7(2):e1001279. doi: 10.1371/journal.ppat.1001279 (PMC3037358; doi:10.1371/journal.ppat.1001279)
Supplement: Table S6 — BAC recombineering strategy. BAC recombineering constructs created by PCR amplifying a selectable cassette (β-lactamase, using pGEM-T Easy as template, primer sequences A) incorporating unique BAC sequences for targeted recombination (sequences B). (0.06 MB DOC) [file ppat.1001279.s011.doc]

**Table S6.** BAC recombineering strategy.

| 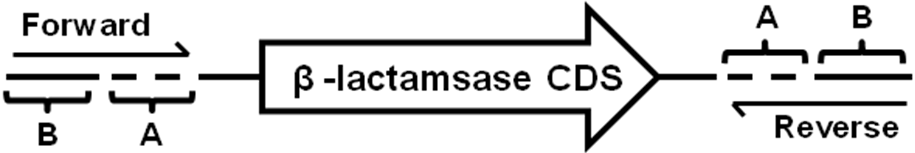 |
| --- |
| **A: selectable cassette specific primer sequences** |
| Forward: 5’-agagttggtagctcttgatc-3’ |
| Reverse: 5’-cattcaaatatgtatccgctc-3’ |
| **B: BAC target specific primer sequences (recombination sites)** |
| Target 1 forward: 5’-cgatttaccctcagcaaatgatagcaacacgttaggtatacgtgctcgtt-3’ |
| Target 1 reverse: 5’-tctatggatactgcgctgcagcagcagcgcgggcatacttagttgctgca-3’ |
| Target 2 forward: 5’-cagtgtatctagtgtatactgaggtgcctatacaccgcaaatagtaaata-3’ |
| Target 2 reverse: 5’-tacctgcctatacaccccaatattgacgaagtgaccactggtcagtttat-3’ |
| Target 3 forward: 5’-tgggagcggtggcagtgtaagtacaccgctgcaccatcagtagctgctgc-3’ |
| Target 3 reverse: 5’-tgtcttaactgcaacagatgtgcgcatgccaagactggcgcctgaattac-3’ |
| Target 4 forward: 5’-taatatgggtgcccgttgtctgccaaagatttggcagtgcagcatggaac-3’ |
| Target 4 reverse: 5’-aacaaaatcgtcaatcccaagtacaacaacggacatcctgtctttcggaa-3’ |
| Target 5 forward: 5’-tacgaacaatgaggacagatattgacgcggttttcttcaggagaaggaat-3’ |
| Target 5 reverse: 5’-ccaatggaccaagccatccatgcacgcgacaggagcattcgagtacctgt-3’ |
| Target 6 forward: 5’-ctgtagtacaatgacaccttcatctcctgaaacatcaaacatacgacaaa-3’ |
| Target 6 reverse: 5’-tggggcctcccccaaaacttacacaatggagcagatacgtttactgtccg-3’ |
| Target 7 forward: 5’-ttcttctacgcgcaggcagacaggcggacccaactggtacacactcgtga-3’ |
| Target 7 reverse: 5’-tgtgagggtgggttctcccgcgtaggctcatgcagctgttcatactggtc-3’ |
| Target 8 forward: 5’-gaatgccattcccttgccaccgattgactaaagtctacgtgaattctgca-3’ |
| Target 8 reverse: 5’-caaataaattcctcaataataatcaatagtcgtcttatttagatgcatgc-3’ |

BAC recombineering constructs created by PCR amplifying a selectable cassette (β-lactamase, using pGEM-T Easy as template, primer sequences A) incorporating unique BAC sequences for targeted recombination (sequences B).
