# Supplementary material for: Genetic Mapping Identifies Novel Highly Protective Antigens for an Apicomplexan Parasite
Source: PLoS Pathog. 2011 Feb 10;7(2):e1001279. doi: 10.1371/journal.ppat.1001279 (PMC3037358; doi:10.1371/journal.ppat.1001279)
Supplement: Table S7 — Validation of targeted BAC recombineering by PCR. Primers designed to confirm targeted disruption: knockout = amplification between target-flanking genomic DNA and the insert, wild-type = amplification between target-flanking genomic DNA and the genomic DNA target. Results shown in Figure S4. (0.04 MB DOC) [file ppat.1001279.s012.doc]

**Table S7.** Validation of targeted BAC recombineering by PCR.

| Target | Insertion result | Forward primer | Reverse primer |
| --- | --- | --- | --- |
| 1 | Knockout | 5’-cagcagcgtaacttggaggaga-3’ | 5’-agcgtcagaccccgtagaaaag-3’ |
|  | Wild-type | As for Target 1 positive | 5’-gctgcggagtcagtgtagctgt-3’ |
| 2 | Knockout | 5’-atcccttctcgtttgctgcatt-3’ | As for Target 1 positive |
|  | Wild-type | As for Target 2 positive | 5’-gctgctgctgctaaggagactg-3’ |
| 3 | Knockout | 5’-gggccccaatagataaggaggt-3’ | As for Target 1 positive |
|  | Wild-type | As for Target 3 positive | 5’-ggtagtggcgcagttattggtg-3’ |
| 4 | Knockout | 5’-atgagcataggatgcaggcgta-3’ | As for Target 1 positive |
|  | Wild-type | As for Target 4 positive | 5’-cttggagttcttgccgatcctt-3’ |
| 5 | Knockout | 5’-ccgaccgaagttttgttcacac-3’ | As for Target 1 positive |
|  | Wild-type | As for Target 5 positive | 5’-gcgggtacaagcagtagaagca-3’ |
| 6 | Knockout | 5’-gaggcggtttcctttatgatgc-3’ | As for Target 1 positive |
|  | Wild-type | As for Target 6 positive | 5’-gtgcttcaaagtcgctgctcac-3’ |
| 7 | Knockout | 5’-gtggagcttttcctggtggagt-3’ | As for Target 1 positive |
|  | Wild-type | As for Target 7 positive | 5’-cgttttcaggtggggcttacat-3’ |
| 8 | Knockout | 5’-ataagggcgacacggaaatgtt-3’ | 5’-tgcaagcatgtgcatccaata-3’ |
|  | Wild-type | 5’-gtggtcgcatatctgcatcagg-3’ | As for Target 7 positive |

Primers designed to confirm targeted disruption: knockout = amplification between target-flanking genomic DNA and the insert, wild-type = amplification between target-flanking genomic DNA and the genomic DNA target. Results shown in **Figure S4**.
