# Supplementary material for: Genetic Mapping Identifies Novel Highly Protective Antigens for an Apicomplexan Parasite
Source: PLoS Pathog. 2011 Feb 10;7(2):e1001279. doi: 10.1371/journal.ppat.1001279 (PMC3037358; doi:10.1371/journal.ppat.1001279)
Supplement: Table S8 — EmaxBAC8f18 and EmaxBAC2k08 specific primers for LD PCR and nested PCR to confirm LD PCR amplicon identity. *Putative annotation. (0.03 MB DOC) [file ppat.1001279.s013.doc]

**Table S8.** *Emax*BAC8f18 and *Emax*BAC2k08 specific primers for LD PCR and nested PCR to confirm LD PCR amplicon identity.

| BAC ID | Target | LD PCR primers | Nested confirmatory primer |
| --- | --- | --- | --- |
| *Emax*BAC8f18 (see **Figure 2**) | Sulphate transporter* | For: 5’-gggcaacagcagtctcacatct-3’ Rev: 5’-caagttgcacccgagtcagtct-3’ | For: 5’-tgtggtattgtaggcggattgg-3’ Rev: 5’-tgcctgaggaccttgcaataaa-3’ |
|  | Apical membrane antigen 1 | For: 5’- ggctttcgtggatctacctgct -3’ Rev: 5’-gtctgcacacctcaacagcaga-3’ | For: 5’-gttcatggacggggtttgtgta-3’ Rev: 5’-ggtcggctatctagcgctttgt-3’ |
|  | Transcription elongation factor* | For: 5’-cgaacacagtctccttgccagt-3’ Rev: 5’-tcctcgattctacctgctgctg-3’ | For: 5’-ccgtctgtgtaccagcctctgt-3’ Rev: 5’-caattcttcaaccttgcgaacg-3’ |
| *Emax*BAC2k08  (see **Figure 3**) | ncRNA* | For: 5’-aagagggaggagggggtacaga-3’ Rev: 5’-ttcgtcaaagcagcagatgaga-3’ | For: 5’-caaacccgtaagcccggtaata-3’ Rev: 5’-tgtttgtttgctcaccgtctga-3’ |
|  | Unknown | For: 5’-taattgccgcaccatacaggtt-3’ Rev: 5’-gagagggagaggtcggtgtgtt-3’ | For: 5’-tcgccgaatctctttggtgtaa-3’ Rev: 5’-tacaagtgggaggtttggagca-3’ |
|  | Repeats + Cyclophilin-RNA interacting protein* | For: 5’-agcaacagcagccatacaatcc-3’ Rev: 5’-atgggcacaagaaaaagggaaa-3’ | For: 5’- tgctgctgcttcatcctcttct -3’ Rev: 5’-gtggtggtggggttgttgtatg-3’ |
|  | SCY kinase related protein* | For: 5’-gcacatgcttgcaaaccctaaa-3’ Rev: 5’-aagctcgggtgtctgtgtcaac-3’ | For: 5’-atgagtgcaatgctgtccgatt-3’ Rev: 5’-tgctctgtctgcacgattgaag-3’ |

*Putative annotation.
